# Gallate-oriented surface engineering of lignin nanoparticles for enhanced antibacterial activity

**DOI:** 10.1016/j.mtbio.2026.103517

**Published:** 2026-07-31

**Authors:** Zonghong Lu, Shujun Liang, Depeendra Yadav, Hao Zhang, Jessica M. Rosenholm, Chunlin Xu, Tapani Viitala, Xiaoju Wang

**Affiliations:** a Laboratory of Natural Materials Technology, Department of Engineering and Information Technology, Åbo Akademi University, Henrikinkatu 2, Turku, FI-20500, Finland; b Pharmaceutical Sciences Laboratory, Department of Natural and Health Sciences, Åbo Akademi University, Tykistökatu 6A, Turku, FI-20520, Finland

**Keywords:** Lignin nanoparticle, Gallate ester, Surface chemistry, Amphiphilic balance, Antibacterial activity, Biocompatibility

## Abstract

In combating bacterial infections, antibacterial nanomaterials offer a non-pharmacological alternative to conventional antibiotics. Biomass-derived lignin exhibits intrinsic antibacterial activity from polyphenolic motifs and can readily self-assemble into nanostructures due to its amphiphilicity. However, the formation of lignin nanoparticles (LigNPs) is governed by a delicate balance of noncovalent interactions, making assembly outcomes highly dependent on lignin source and fractionation process and thereby limiting precise control over the surface chemistry and functional-group presentation. Here we have developed a gallate-oriented surface engineering strategy that combines boronated lignin nanoparticles (BLigNPs) core with gallate derivatives (GDs) corona, enabling tunable surface chemistry and modulating their bactericidal activity to preferentially target Gram-negative or Gram-positive bacteria. Through spatially grafting redox-active pyrogallol moieties in a tannic acid (TA) corona, BLigNP-TA exhibited the highest antibacterial activity against *S. aureus*. In contrast, owing to the balanced hydrophilicity/hydrophobicity in a propyl gallate (PG) corona, BLigNP-PG showed the highest antibacterial activity against *E. coli*. Consistent with this interpretation, BLigNP-PG exhibited enhanced envelope association and permeabilization in both Gram-negative and Gram-positive bacteria, supporting a contact-associated antibacterial action via a multimodal and multitarget mechanism. Beyond planktonic bacteria, both corona designs enhance the nanoparticle association with biofilm-relevant interfaces and facilitate the nanoparticle transport within biofilms. Notably, BLigNP-PG exhibited good biocompatibility with fibroblasts and negligible toxicity in zebrafish, underpinning its translational potential as an antibacterial nanomaterial with high selectivity toward bacteria over mammalian cells.

## Graphical abstract

**Figure:**
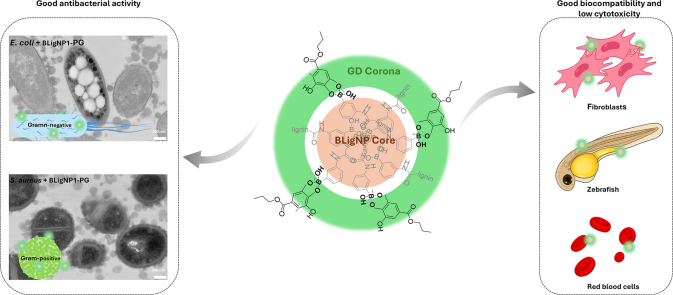

## 1 Introduction

The rising prevalence of infections caused by multidrug-resistant bacteria poses a significant global health threat [1]. Biofilm formation is initiated by bacterial adhesion and is driven by the production of extracellular polymeric substances (EPS) that stabilize the surface-associated bacterial communities [2]. The resulting 3D architecture functions as a physical barrier that shields embedded bacteria from the host immune response and limits the efficacy of conventional antibacterial agents, while persister cells and pronounced spatial and chemical heterogeneity in biofilm further contribute to antibiotic tolerance [3,4]. As a matter of fact, the preclinical pipeline for new antibiotics is slow and fragile, and this necessitates alternative, non-pharmacological approaches to combat biofilm-associated infections [5]. Innovative developments in nanotechnology have enabled antibacterial approaches that show strong promise not only in efficiently suppressing the growth of biofilms but also in eradicating established biofilms [4]. On the nanoscale, the high surface-area-to-volume ratio of nanoparticles (NPs) allows high reactivity and dynamic interactions with both pathogens and the host [[6], [7], [8]]. They can either enhance the effectiveness of existing antibacterial agents as drug carriers or serve as standalone therapeutics with intrinsic antibacterial activity [9]. The diverse mechanisms by which NPs interact with bacteria and disrupt in biofilms encompass the disruption of the cell wall and membrane, generation of reactive oxygen species (ROS), and damage to intracellular components [4,7]. Engineered noble metals (Ag and Au) and metal oxides (Fe_3_O_4_, CuO, and MnO_2_) NPs are emerging as enzyme-mimicking nanomaterials that effectively generate ROS to kill bacteria via multimodal mechanisms [10,11]. Polymeric NPs based on poly(amino-acid) and self-assembled cationic polymers, inspired by host defence peptides, have also shown promise as alternatives to conventional antibiotics [[12], [13], [14]]. Careful engineering of the physicochemical properties of NPs, including size, shape, and surface chemistry (corona composition) is pivotal for tailoring antibacterial performance and their ability to penetrate biofilms and interact with embedded bacterial cells [15]. However, optimizing these properties to enhance antibacterial activity may also increase cytotoxicity, as the same mechanisms that kill pathogens may disrupt mammalian membranes and interact with host biomacromolecules [16]. Therefore, the rational design of NPs across diverse platforms increasingly emphasizes selective antibacterial modalities that enable effective pathogen eradication while minimizing collateral damage to mammalian cells.

Naturally derived polyphenol-rich biopolymers have garnered widespread interest in bionanotechnology as building blocks for antibacterial nanomaterials [[17], [18], [19], [20]]. Lignin, the most abundant aromatic biopolymer on Earth, is ubiquitous in vascular plants and is biosynthesized through non-template oxidative radical polymerization of three primary monolignols, which generates heterogeneous distributions of hydroxyphenyl (H), guaiacyl (G), and syringyl (S) units and diverse interunit linkages [21,22]. Moreover, its molecular architecture is further shaped by lignin source and biomass fractionation process, which further contribute to the structural heterogeneity in this polyphenolic biopolymer [23]. These structural features and functional-group distributions are key determinants of physicochemical properties and bioactivity of lignin, particularly its antibacterial activity, which is associated with phenolic hydroxyl and methoxy functionalities [24]. Its amphiphilic nature also enables self-assembly into nanostructures upon changes in solubility. Nanoprecipitation has been widely used to prepare lignin NPs (LigNPs), with common approaches including antisolvent exchange, pH-shifting by acids, or ultrasonication [25,26]. Among these, antisolvent precipitation is the most effective approach to achieve spherical LigNPs with uniform size and smooth surfaces [25]. To date, research has focused on how the lignin macromolecular architecture governs the shape and size of LigNPs, as well as their surface chemistry and topography, and how these NP attributes dictate the performance as both intrinsically antibacterial nanomaterials and drug nanocarriers [27,28]. Moreover, hybrid lignin-metal nanocomposites are widely employed to enhance antibacterial activity, as phenolic functionalities in lignin can act as reducing and capping agents for certain metals or metal oxides [29,30]. Representative state-of-the-art systems include the LigNP@Ag, which consists of monodisperse spherical LigNP (∼120 nm) decorated with surface-anchored AgNP (∼10 nm), as well as TeLigNP, which comprises irregularly shaped nanocomposites (100–200 nm) consisting of a lignin matrix hosting embedded tellurium nanoclusters (∼10 nm) [31,32]. Despite their strong antibacterial activities, these systems predominantly rely on the release of metal ions to exert antibacterial effects, which may limit their biosafety and long-term efficacy, particularly in the context of resistance development [33,34]. Back to NP engineering, surface chemistry (corona composition) plays a critical role in endowing LigNPs with high selectivity as intrinsically antibacterial nanomaterials [35]. The amphiphilicity-driven self-assembly of polyphenolic lignin enables the formation of LigNP, however, this process lacks a precise control over the surface chemistry owing to the complex and heterogeneous macromolecular structure of lignin [36]. Systematic comparisons of LigNPs from different sources and fractionation processes remain limited, particularly their surface chemistries and functionalities that govern contact-mediated interactions with bacteria and biofilms.

Gallic acid (GA), a naturally occurring trihydroxybenzoic acid found in plant tannins, represents a simple and well-defined phenolic motif [37]. Its pyrogallol-type structure provides adjacent phenolic hydroxyl groups that endow GA and its gallate derivatives with redox activity, metal-chelating capacity, and antibacterial relevance [38]. Importantly, these adjacent hydroxyl groups can also form dynamic boronate ester interactions with phenylboronic acid moieties, offering a rational route for metal-free surface engineering of boronated lignin nanoparticles. Although GA is not a native lignin structural unit, it shares key aromatic phenolic features with lignin and can introduce tannin-like interfacial chemistry onto lignin nanoparticles [39]. Accordingly, we designed a surface-engineering strategy using wheat-straw alkaline lignin (WSAL)-derived nanoparticles and a benchmark library of gallates, together with tannic acid, to tune corona composition and systematically regulate nanoparticle surface chemistry. This all-polyphenolic architecture, comprising a lignin core and a gallate/tannin corona, is hypothesized to enhance NP interactions with bacteria and the host. Our aim is to elucidate the relationships between surface chemistry and bactericidal activity of the gallate-engineered LigNPs, thereby enabling the rational design of selective antibacterial nanomaterials from naturally derived polyphenolic biopolymers. The current research thus encompasses the chemical design for all-polyphenolic NPs with multi-instrumental characterization to precisely define the nanoscale dimensions and surface functionalities of the nanomaterials. The amphiphilicity and redox activity of these gallate-engineered LigNPs were systematically investigated to correlate the surface chemistry and molecular topography with antibacterial activity using quantitative microbial bioassays. Their performance was further assessed under biofilm-relevant conditions through biofilm inhibition and eradication assays. Furthermore, multi-parametric surface plasmon resonance (MP-SPR) was employed to analyze the dynamic association of LigNPs with the biofilm-embedded bacteria, providing real-time quantitative information. Cytotoxicity was evaluated *in vitro* in mammalian cell cultures, and biocompatibility was assessed *in vivo* in zebrafish models. Overall, the results indicate that surface-engineered gallate motifs enhance antibacterial activity through multimodal contact-dependent mechanisms, including ROS generation, membrane potential disruption, and leakage of intracellular contents upon NP–bacterium contact.

## 2 Materials and methods

### 2.1 Materials and chemicals

Wheat straw alkaline lignin (WSAL) was supplied by CH Bioforce Oy (Espoo, Finland) and obtained from wheat straw via the BLN biorefinery process. Gallic acid (GA), methyl gallate (MG), ethyl gallate (EG), propyl gallate (PG), and tannic acid (TA) were purchased from Sigma-Aldrich. 3-Aminophenylboronic acid hydrochloride (APBA), 4-(4,6-dimethoxy-1,3,5-triazin-2-yl)-4-methylmorpholinium chloride (DMTMM), γ-valerolactone, dimethyl sulfoxide (DMSO), resazurin, glutaraldehyde, paraformaldehyde, and 3,4-dichloroaniline were also obtained from Sigma-Aldrich. 2,2′-azino-bis (3-ethylbenzothiazoline-6-sulfonic acid) diammonium salt (ABTS) was purchased from VWR, and 2,2-diphenyl-1-picrylhydrazyl (DPPH) was purchased from TCI Europe. Luria-Bertani (LB) broth and agar powder, tryptic soy (TS) broth and agar powder, Dulbecco's modified Eagle medium (DMEM, high glucose, GlutaMAX™ Supplement), SYTO 9 and propidium iodide (PI) stains, Fetal bovine serum (FBS), penicillin**–**streptomycin (Pen**–**strep), and the Coomassie (Bradford) protein assay kit (including bovine serum albumin, BSA standards) were supplied by Thermo Fisher Scientific. Sheep blood in Alsever's solution was purchased from TCS Biosciences. Cell Counting Kit-8 (CCK-8), Calcein AM and EthD-III, 2',7'-dichlorodihydrofluorescein diacetate (DCFH-DA), and DiBAC_4_(3) were purchased from MedChemExpress. *Escherichia coli* (*E. coli*, ATCC 25922) and *Staphylococcus aureus* (*S. aureus*, ATCC 25923) were obtained from the American Type Culture Collection (ATCC). Human dermal fibroblasts (HDFs, Cat. No. C0135C) were purchased from Thermo Fisher Scientific and used at passages 7–8. All solvents and chemicals were used as received without further purification.

### 2.2 Preparation of boronated lignin nanoparticles (BLigNPs)

WSAL was used as the starting material to prepare phenylboronic-acid-functionalized lignin, which was subsequently converted into BLigNPs by antisolvent precipitation. Briefly, WSAL (100 mg) was dissolved in a mixture of γ-valerolactone (7 mL) and MES buffer (3 mL, 0.1 M, pH 5.0). DMTMM (18 mg) was then added to activate carboxyl groups on lignin, and the reaction mixture was stirred at 500 rpm for 1 h.

Next, 8 mg of APBA was added, and the mixture was continuously stirred for 24 h. The resulting solution was subsequently injected into DI water (100 mL) under vigorous stirring (1000 rpm) to induce lignin NPs formation, and stirring was continued for an additional 1 h. The dispersion was transferred to dialysis tubing (molecular weight cut-off 3500 Da) and dialyzed against DI water to remove γ-valerolactone, yielding BLigNP1.

BLigNP2 and BLigNP3 were prepared using the same procedure, with DMTMM/APBA amounts of 36 mg/16 mg and 54 mg/24 mg, respectively.

### 2.3 Preparation of gallate derivatives (GDs) functionalized BLigNP (BLigNP-GDs)

BLigNPs dispersion (10 mg mL^−1^) was mixed with various GD ligands (25 μM) and stirred for 1 h. Subsequently, 0.02 M PBS (pH 7.4) was added dropwise at room temperature, and the mixture was stirred continuously overnight to complete surface functionalization. The resulting suspension was centrifuged (10000 × g) for 10 min at 4 °C to remove unreacted GD monomers. Finally, the pellet was resuspended and stored in 0.01 M PBS (pH 7.4).

### 2.4 Characterization

The nanoscale dimensions and surface functionalities of BLigNPs and BLigNP-GDs characterized by Z-average size, zeta potential, molar mass analysis, FTIR, TEM, ^11^B NMR, and ^31^P NMR. Their antibacterial activity was evaluated by colony counting, growth curve analysis, and biofilm inhibition and eradication assays Nanoparticle–bacterium interactions were further investigated by TEM, SEM, CLSM, and MP-SPR. Cytotoxicity and biosafety were assessed by CCK-8 assay in HDFs, hemolysis assay in red blood cells, and locomotor activity analysis in zebrafish. In addition, antibacterial mechanism-related properties were examined through water contact angle, surface tension, antioxidant activity, ROS generation, membrane potential, and protein leakage measurements. Detailed experimental information is provided in the Supplementary Information.

## 3 Results

### 3.1 Synthesis and characterization of BLigNPs BLigNP-GDs

**Fig. 1:**
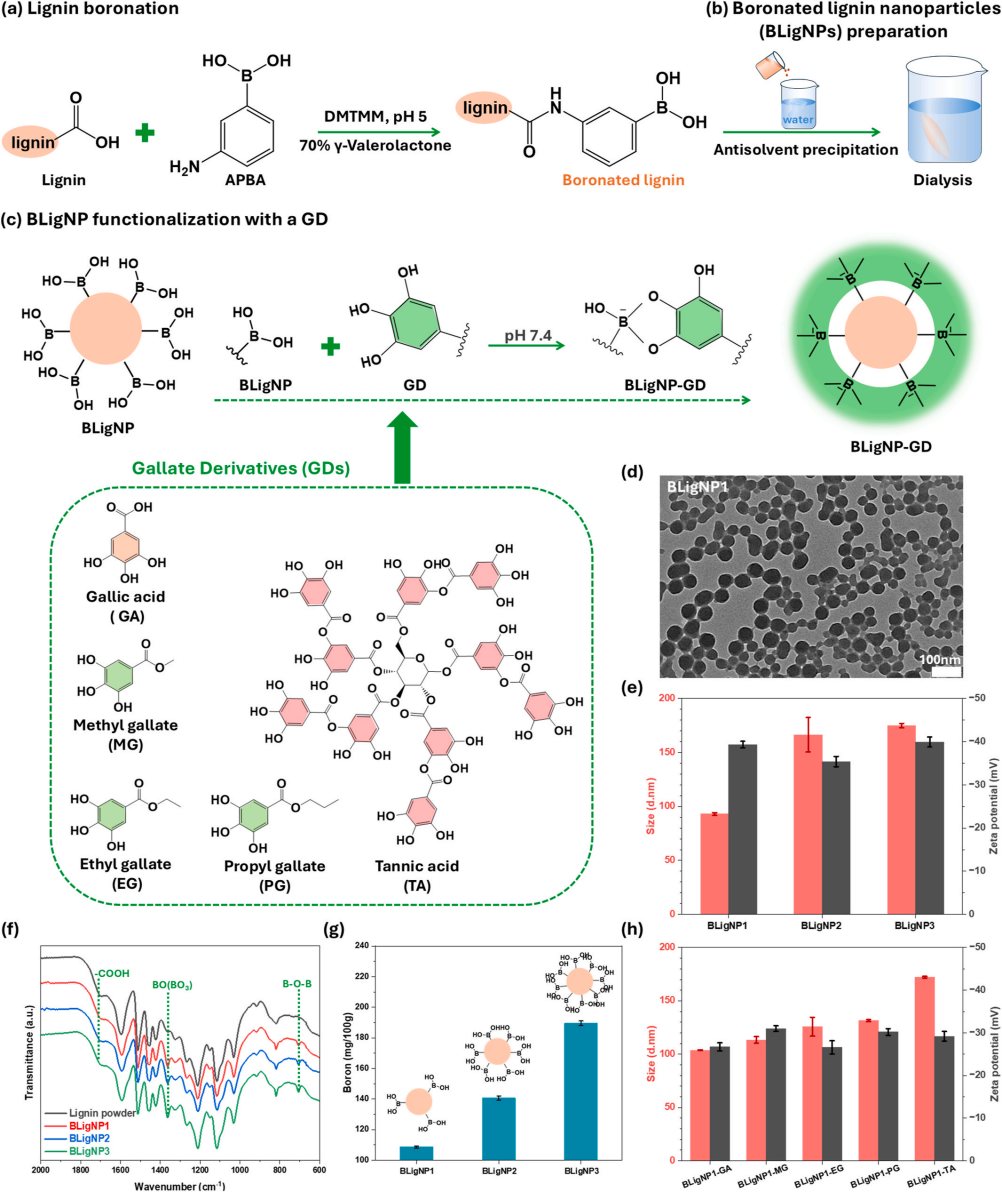
Design strategy for the fabrication and characterization of all-polyphenolic NPs. (a, b) Schematic illustration of BLigNP preparation via DMTMM-mediated amidation of lignin with aminophenylboronic acid (APBA) under mildly acidic conditions, followed by antisolvent precipitation and dialysis to remove unreacted reagents and obtain an aqueous BLigNP dispersion. (c) Fabrication of BLigNP-GDs by grafting various GDs onto the surface of BLigNP in PBS. (d) TEM image of the BLigNP1. (e) Hydrodynamic diameter and zeta potential in PBS (0.1 M, pH 7.4) of BLigNPs measured by dynamic light scattering (DLS) and electrophoretic light scattering (ELS), respectively. (f) ATR-FTIR spectra of dried pristine lignin powder and BLigNPs. (g) Boron content in BLigNPs measured by ICP-OES. (h) Hydrodynamic size and zeta potential of BLigNP-GDs determined by DLS and ELS, respectively.

As illustrated in Fig. 1a–c, the platform for synthesising all-polyphenolic NPs involves three sequential steps: (a) preparing boronated lignin by grafting APBA via amidation; (b) fabricating BLigNPsthrough antisolvent nanoprecipitation; (c) engineering the corona composition of BLigNPs with GDsvia the reversible boronate-ester formation between phenylboronic acid and pyrogallol. A benchmark library comprising GA, three alkyl gallates, and TAwas screened to modulate the surface chemistry, including the surface-presented functional groups, interfacial hydrophilic-hydrophobic balance, and molecular multivalency. The density of available surface anchoring sites was primarily governed by the extent of phenylboronic acid incorporation into lignin.

In detail, an alkaline lignin derived from wheat straw was used as the starting material, and APBA was covalently grafted onto its carboxyl groups through DMTMM-mediated amide formation (Fig. 1a). By adjusting the dosages of both APBA and the coupling reagent DMTMM, the pristine lignin was functionalized with tunable grafting densities of phenylboronic acid group and subsequently assembled into BLigNPs via antisolvent-induced self-assembly (Fig. 1b), yielding uniform nanospheres (Fig. 1d and Fig. S5). Moreover, increasing the grafting density of phenylboronic acid group led to a progressive increase in hydrodynamic diameter, while the zeta potential shifted to more negative values (Fig. 1e). All formulations exhibited zeta potentials more negative than −30 mV, consistent with substantial electrostatic repulsion and good colloidal stability under the tested conditions. Successful grafting of phenylboronic acid groups onto lignin was evidenced by FTIR spectroscopy, which exhibited characteristic bands attributable to B-O stretching in BO_3_ units and B-O-B vibrations at 1364 and 707 cm^−1^, respectively (Fig. 1f), in agreement with previous reports [40,41]. Inductively Coupled Plasma-Optical Emission Spectroscopy (ICP-OES) results showed that the boron content associated with grafted phenylboronic acid group increased from 108.7 mg per 100 g for BLigNP1 to 189.5 mg per 100 g for BLigNP3 (Fig. 1g). Moreover, SEC-MALS revealed an increased molar mass for BLigNPs compared with pristine lignin (Table S1), while ^31^P NMR showed a reduced carboxyl groups content (Table S2), providing additional evidence for successful grafting of phenylboronic acid groups. BLigNP formation is proposed to proceed through antisolvent-induced desolvation, nucleation, and particle growth [36]. When boronated lignin dissolved in aqueous γ-valerolactone is rapidly introduced into excess water, the resulting decrease in solvent quality weakens lignin-solvent interactions and promotes the association of poorly solvated aromatic domains through hydrophobic interactions, π–π stacking, hydrogen bonding, and van der Waals forces [42,43]. The resulting nanoscale nuclei subsequently grow through further lignin association, whereas surface-accessible ionizable and relatively hydrophilic groups contribute to colloidal stabilization. On this basis, the introduced phenylboronic acid groups, owing to their relatively hydrophilic nature, are expected to be preferentially enriched on the surface of the BLigNPs. This interpretation is supported by the liquid-state ^11^B NMR spectra of BLigNPs dispersed in D_2_O (Fig. S6), which primarily probes solvent-accessible (surface-exposed) boron. These accessible phenylboronic acid groups subsequently serve as reactive anchoring sites for boronate ester formation with GDs, thereby enabling post-assembly modulation of the nanoparticle corona composition (Fig. 1c).

BLigNP1, which has the lowest boron content, was representatively selected to investigate the role of surface chemistry of GD corona in modulating antibacterial activity. This modulation can be approached through two molecular design strategies: (i) tuning surface amphiphilicity (hydrophilicity/hydrophobicity) balance to optimize interactions with and permeation through the bacterial membrane, and (ii) incorporating polyphenol-rich architectures in which multiple spatially distributed phenolic hydroxyl groups facilitate multivalent binding to bacterial surface biomacromolecules (*e.g.*, proteins and cell-wall components) and metal coordination, thereby enhancing antibacterial efficacy through membrane disruption and ROS generation. GA is 3,4,5-trihydroxybenzoic acid bearing a free carboxylic acid group. Its ester derivatives, including MG, EG, and PG, are formed by esterification of the carboxyl group with alkyl chains of progressively increasing length. These gallate moieties can be grafted onto the surface of BLigNPs via boronate-diol complexation, with the alkyl chains preferentially orienting outward to finely tune the amphiphilic balance. Strategically, the sequence from BLigNP-MG to BLigNP-EG, and to BLigNP-PG represents an increased hydrophobicity in the engineered corona composition of gallate esters. Meanwhile, TA is a polyphenolic polygalloyl glucose composed of multiple GA units esterified to a glucose core, forming a globular architecture with spatially distributed pyrogallol-type motifs that can be presented on the surface of NP. In this molecular design, the BLigNP-TA *vs.* BLigNP-GA comparison to some extent reflects the modulation of molecular topography. For BLigNP-TA, the TA ligand contains a spatial distribution of multiple GA units at one anchoring site; and for BLigNP-GA as a reference, it contains only monomeric GA motifs conjugated onto BLigNP surface. The surface functionalization of GDs on BLigNP1 is supported by a shift in zeta potential in PBS at pH 7.4 and, more notably, an increase in hydrodynamic size (Fig. 1h and Fig. S8). Along the sequence of BLigNP-MG, -EG, and -PG, a decrease of zeta potential at the same condition was confirmed as the hydrophobicity increased. BLigNP-TA showed the largest hydrodynamic diameter but the lowest negative zeta potential among all the BLigNP-GDs.

### 3.2 *In vitro* antibacterial activity of BLigNP-GDs

**Fig. 2:**
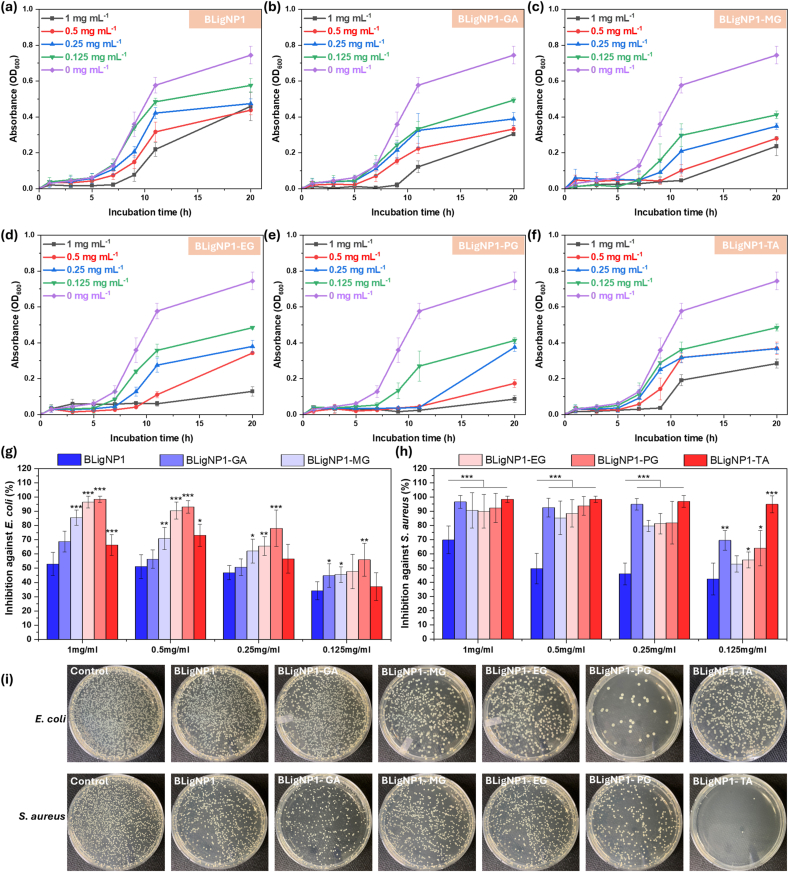
In vitro antibacterial activity of BLigNP1-GDs. (a–f) Growth curves of *E. coli* upon incubation with different BLigNP1-GDs at various concentrations (0–1 mg mL^−1^). Growth inhibition of (g) *E. coli* and (h) *S. aureus* after 15 h incubation with BLigNP1-GDs at various concentrations (0–1 mg mL^−1^). (i) Digital images of agar-plate showing reduced colony formation after treatment with BLigNP1 and BLigNP1-GDs at 1 mg mL^−1^. Data are presented as the mean ± SD (n = 3), *p < 0.05, **p < 0.01, ***p < 0.001.

The *in vitro* antibacterial performance of BLigNP1-GDs was evaluated against Gram-negative *Escherichia coli* (*E. coli*) and Gram-positive *Staphylococcus aureus* (*S. aureus*) by recording time-course OD600 values to generate growth curves at different BLigNP1-GDs concentrations. While the growth inhibition of *E. coli* (Fig. 2a–f) and *S. aureus* (Fig. S9) increased with increasing BLigNP1-GDs concentration, differences in antibacterial activity between surface functionalization were more pronounced when samples were compared at the same concentration. At 1 mg mL^−1^, BLigNP1 functionalized with alkyl gallates (MG/EG/PG) exhibited strong inhibition against *E. coli*, as demonstrated by consistently lower OD600 values than BLigNP1 and the GA-/TA-functionalized BLigNPs. Meanwhile, BLigNP1-TA showed the greatest suppression of *S. aureus* growth among the tested samples. These kinetic trends were then validated by enumeration of colony-forming units (CFUs) on agar plates (Fig. 2i), which provides a direct measure of viable bacteria. As a baseline, BLigNP1 reduced viable *E. coli* CFUs by 53.0% at 1 mg mL^−1^, whereas this reduction decreased to 34.2% at 0.125 mg mL^−1^ (Fig. 2g). Upon surface functionalization, the activity against *E. coli* becomes strongly dependent on the GDs species. Using BLigNP1 as the reference sample, BLigNP1 functionalized with alkyl gallates showed a progressively stronger inhibition at a given concentration (BLigNP1-MG < BLigNP1-EG < BLigNP1-PG), indicating that increasing alkyl-chain length in the corona enhances the antibacterial activity against *E. coli*. Notably, it has been previously reported that soluble alkyl gallates exhibited higher antibacterial activity with increasing alkyl-chain length (up to an optimal, as reported) [[44], [45], [46], [47]]. Our finding is also consistent with what was reported on pectin-PG conjugates, where PG was grafted onto pectin via esterification between the phenolic OH groups of PG and the carboxyl groups of pectin, thereby exposing the alkyl chain toward the outer interface and enhancing growth inhibition against both *E. coli* and *S. aureus*, with a more pronounced effect against *E. coli* than that observed for pectin-GA [48]. BLigNP1-PG exhibited the highest inhibition (98.5%) against *E. coli* at 1 mg mL^−1^. In contrast, BLigNP1-GA (68.7%) and BLigNP1-TA (66.3%) showed relatively low inhibition at the same concentration, indicating only minimal improvement relative to the reference BLigNP1. For *S. aureus*, BLigNP1-TA achieved the highest inhibition (98.4%) at 1 mg mL^−1^ and maintained strong activity (94.9% inhibition) even at 0.125 mg mL^−1^. Notably, BLigNP1-PG also reached 92.3% inhibition against *S. aureus* at 1 mg mL^−1^ but dropped to only 64.1% inhibition at 0.125 mg mL^−1^ (Fig. 2h). These results clearly demonstrate that BLigNP-GDs exhibit species specificity governed by the engineered corona surface chemistry.

To further study how the grafting density of GDs influences the antibacterial performance, a series of BLigNPs with different densities of phenylboronic acid groups were prepared and used as cores for subsequent functionalization with GDs. Based on the antibacterial results above, PG and TA were selected as representative GDs for the following study. The corresponding hydrodynamic size and zeta potential of BLigNPs and BLigNP-GDs are shown in Fig. 1e and h and Fig. S10. Increasing the grafting density of phenylboronic acid groups on the BLigNPs enhanced growth inhibition against both *E. coli* and *S. aureus* (Fig. S11 and Fig. S12). This enhancement can be attributed to the inherent antibacterial activity of phenylboronic acid groups, which may strengthen interactions with bacterial membranes or interfere with cellular processes, as supported by previous studies [49,50]. Surprisingly, varying the surface grafting density of PG or TA on BLigNPs did not lead to significant differences in growth inhibition for either *E. coli* or *S. aureus*. This observation is consistent with a plateau in activity, suggesting that a critical surface density of functional groups may be sufficient to achieve near-maximal growth inhibition. Beyond this level, additional grafting may not further enhance antibacterial performance, potentially because of steric crowding, reduced accessibility of surface ligands, and/or saturation of binding sites on the bacterial envelope. This highlights that the specific chemical functionality of grafted groups and the availability of active groups may be more influential than the overall surface density, consistent with observations reported in other surface-engineered antibacterial systems [51]. Moreover, after 6 months of storage, BLigNP1, BLigNP1-PG, and BLigNP1-TA showed only slight changes in hydrodynamic diameter and zeta potential, corroborating a confirmed long-term colloidal stability (Fig. S13). Notably, bacterial metabolic activity (*E. coli* and *S. aureus*) in the presence of these NPs increased modestly over the storage period, yet remained significantly lower than the control, demonstrating their long-term stability of antibacterial activity (Fig. S14).

### 3.3 Inhibition of bacterial biofilm formation and eradication of bacterial biofilms

Biofilms are structured bacterial communities embedded in a self-produced EPS matrix, typically composed of polysaccharides, proteins, and extracellular DNA [52]. This matrix functions as a protective shield that enables bacterial survival under harsh environmental conditions (*e.g.*, antibiotic exposure) and is a major driver of drug tolerance and recurrent infections [8]. Based on the planktonic antibacterial results, BLigNP1-PG and BLigNP1-TA were selected for antibiofilm studies due to their strong inhibition against *E. coli* and *S. aureus*, respectively (Fig. 2). This section quantitatively compares the antibiofilm activities of BLigNP1, BLigNP1-PG, and BLigNP1-TA against representative Gram-negative *E. coli* and Gram-positive *S. aureus* under two settings: biofilm inhibition (inhibition of biofilm formation) and biofilm eradication (treatment of pre-established biofilms).

**Fig. 3:**
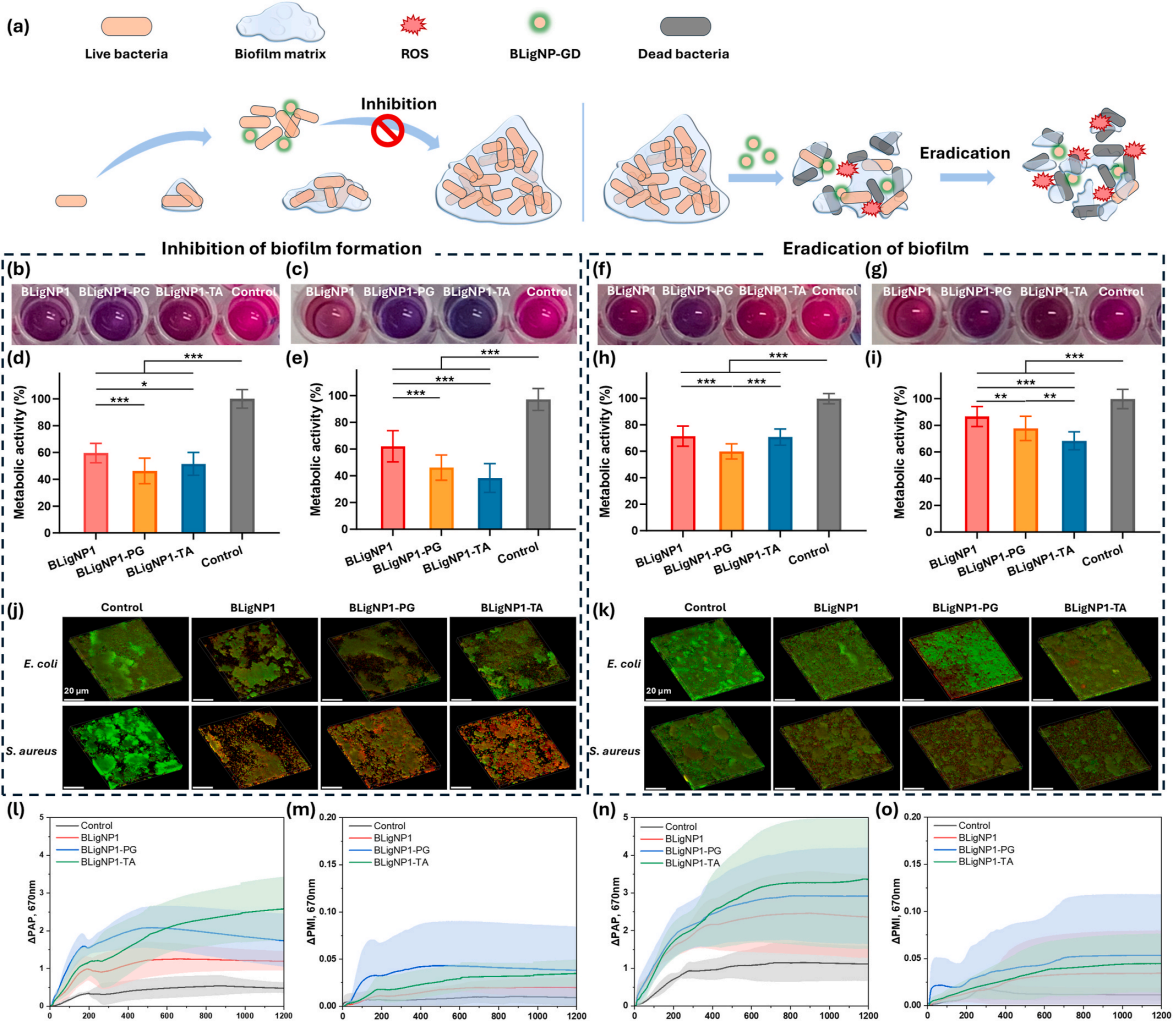
Inhibition of biofilm formation and eradication of pre-established biofilms by BLigNP1 and BLigNP1-GDs at 1 mg mL^−1^. (a) Schematic diagrams illustrating how BLigNPs and BLigNP-GDs inhibit biofilm formation and eradicate pre-established biofilms. Biofilm formation inhibition: representative resazurin-stained images of (b) *E. coli* and (c) *S. aureus* biofilms formed in the presence of BLigNPs and BLigNP-GDs, and corresponding quantification of relative biofilm metabolic activity by the resazurin assay for (d) *E. coli* and (e) *S. aureus*. Biofilm eradication: representative resazurin-stained images of performed (f) *E. coli* and (g) *S. aureus* biofilms after treatment with BLigNPs and BLigNP-GDs, and corresponding quantification of relative biofilm metabolic activity by the resazurin assay for (h) *E. coli* and (i) *S. aureus*. CLSM images of *E. coli* and *S. aureus* biofilm showing (j) inhibition of biofilm formation and (k) eradication of pre-established biofilm following treatment with BLigNP and BLigNP-GDs, assessed by live/dead assay (live cells: green; dead cells: red). Average real-time MP-SPR responses showing changes in (l) ΔPAP and (m) ΔPMI during exposure of *E. coli* biofilm-coated sensor surfaces to different NPs. Average real-time MP-SPR responses showing changes in (n) ΔPAP and (o) ΔPMI during exposure of *S. aureus* biofilm-coated sensor surfaces to different NPs. Data are presented as the mean ± SD (n = 3), *p < 0.05, **p < 0.01, ***p < 0.001. (For interpretation of the references to colour in this figure legend, the reader is referred to the Web version of this article.)

First, biofilm inhibition was assessed by co-incubating bacteria with BLigNP1, BLigNP1-PG, and BLigNP1-TA during biofilm development, followed by quantifying metabolic activity using the resazurin assay (Fig. 3b–e). For both *E. coli* and *S. aureus*, co-incubation with polyphenolic NPs significantly reduced metabolic activity relative to the untreated control, indicating impaired biofilm formation. As expected, BLigNP1-PG showed the lowest metabolic activity during *E. coli* biofilm formation (Fig. 3d). In contrast, in the case of *S. aureus* biofilm inhibition, BLigNP1-TA demonstrated the lowest metabolic activity (Fig.s 3e). The observed species-specific differences in antibiofilm performance between *E. coli* and *S. aureus* can be attributed to differences in cell envelope architectures and associated surface chemistries. During biofilm formation, polyphenolic NPs can directly interfere with bacterial adhesion and early microcolony development, as well as early EPS formation, when bacterial cells remain relatively accessible and the outcome is dominated by direct NP–cell surface interactions. As a Gram-negative bacterium, *E. coli* possesses an outer membrane with a phospholipid inner leaflet and an outer leaflet enriched in lipopolysaccharide (LPS), which functions as a selective permeability barrier and strongly influences the initial adhesion and membrane association of polyphenolic NPs [53]. The balanced amphiphilicity introduced by PG functionalization likely enhances the affinity of the NPs for the phospholipid domains of the outer membrane, facilitating membrane insertion and permeability and thereby promoting biofilm disruption [38]. In contrast, *S. aureus*, a Gram-positive bacterium, lacks an outer membrane but possesses a thick peptidoglycan cell wall enriched in teichoic acids [53]. The hydroxyl-rich hydrophilicity and high density of pyrogallols in TA may promote multivalent noncovalent interactions with the cell envelope (*e.g.*, hydrogen bonding and π–π/hydrophobic interactions), thereby compromising the integrity of the cell wall and cytoplasmic membrane [54].

Next, biofilm eradication was assessed by treating pre-established mature biofilms with the all-polyphenolic NPs and quantifying biofilm metabolic activity using the resazurin assay (Fig. 3f–i). Similar to the biofilm inhibition results, BLigNP1-PG led to the lowest metabolic activity for *E. coli* biofilms, whereas BLigNP1-TA was most effective against *S. aureus* biofilms. When targeting pre-established biofilms, bacterial cells are embedded in a dense EPS matrix that acts as both a diffusion barrier and a sequestration sink, thereby limiting NP penetration and trapping a substantial fraction of the dose in the outer biofilm layers [55]. Accordingly, eradication efficacy depends not only on the intrinsic activity of polyphenolic NPs against biofilm-embedded bacteria, but also on biofilm-scale transport and accessibility, including the ability to reach the biofilm interface, and to penetrate and diffuse within the EPS-rich mature matrix [2]. From a physical transport perspective, penetration into the EPS network becomes increasingly limited for particles larger than ∼200 nm, as the pore architecture of the matrix imposes steric hindrance that restricts their diffusion [56]. Even when particle sizes are geometrically permissive relative to reported pore diameters (∼0.3−2.7 μm), hydrophobic and electrostatic interactions can still modulate the effective diffusivity of particles within the EPS network [57]. In our system, polyphenolic NPs exhibit an average hydrodynamic diameter of ∼100 nm (compared to their size in dry state, approx. 50 nm as determined by TEM, Fig. 1d), which is well below the reported pore-size range, suggesting that matrix penetration is physically feasible. However, the extent and depth of penetration are ultimately governed by polyphenolic NPs surface chemistry and specific interactions with EPS components rather than size alone.

To connect eradication outcomes with real-time interfacial behavior, polyphenolic NP interactions with mature biofilms were further examined by dual-wavelength MP-SPR (Fig. 3l−**o** and Fig. S15). In this approach, the peak angular position (ΔPAP) predominantly reports changes in the effective refractive index and mass loading within the evanescent sensing region, whereas the peak minimum intensity (ΔPMI) is particularly sensitive to optical losses and heterogeneity (*e.g.*, scattering/absorption contributions from rough, microstructure, or particle-laden layers) [58]. For *E. coli* biofilms, exposure to all polyphenolic NPs elicited a rapid initial ΔPAP response, followed by a progressive signal increase during the first 0−200 min at both 670 nm (Figs. 3l) and 785 nm (Fig. S15a), consistent with fast interfacial association. BLigNP1-PG exhibited the largest initial ΔPAP shift and the strongest increase in ΔPMI, indicating that PG functionalization not only accelerates initial association but also induces early-stage microstructural heterogeneity within the optically sensed zone (Fig. 3m and Fig. S15b). Thereafter, both ΔPAP and ΔPMI gradually decreased to a slightly lower quasi-steady level, which may indicate partial loss and/or compaction of material within the evanescent-field sensing region. In contrast, BLigNP1-TA showed more moderate but continuously increasing ΔPAP values throughout the monitoring period, accompanied by only minor changes in ΔPMI, suggesting slower NP accumulation within the established biofilm and comparatively limited structural perturbation. These signatures are consistent with the highly hydrated, polysaccharide-rich extracellular matrix of Gram-negative *E. coli* biofilm and negatively charged bacterial cell surfaces [59]. Given that eradication of mature *E. coli* biofilms is often limited by NP transport through the EPS matrix and access to biofilm-embedded cells, the balanced amphiphilicity of PG-functionalized NPs may promote more favorable partitioning and distribution within hydrated EPS networks, thereby increasing the probability of productive contact with the bacterial envelope and contributing to their enhanced eradication efficacy against *E. coli* biofilm. By contrast, *S. aureus* biofilms are typically more cohesive and diffusion-restrictive, with a matrix enriched in polysaccharides, peptidoglycan, teichoic acids, surface proteins, and eDNA [60]. Consistent with transport being more strongly constrained in such matrices, BLigNP1-TA exhibited the largest sustained ΔPAP at both wavelengths (Fig. 3n and Fig. S15c), indicating stronger accumulation/retention at the NP–biofilm interface within the sensor-proximal region. In parallel, BLigNP1-PG generated the most prominent and continuously increasing ΔPMI response (Fig. 3o and Fig. S15d), which is consistent with progressive development of an optically lossy and heterogeneous near-surface layer driven by sustained restructuring and/or particle-enriched microdomain formation. Importantly, despite these distinct interfacial signatures, BLigNP1-PG and BLigNP1-TA displayed nearly indistinguishable eradication efficacy against *S. aureus* biofilms (Fig. 3i), suggesting that retention-dominated (BLigNP1-TA) and restructuring-dominated (BLigNP1-PG) interactions can converge to a similar net reduction in biofilm viability or metabolic activity. Mechanistically, the high density of phenolic hydroxyl groups on TA-functionalized NPs is expected to promote strong multivalent binding to proteinaceous components of the EPS matrix via hydrogen bonding and hydrophobic/π–π interactions, consistent with widely reported tannin-protein interactions [61,62]. Such interactions would enhance interfacial retention and may locally perturb matrix cross-linking. Conversely, PG functionalization is more consistent with a restructuring-dominant mode of action, as reflected by the monotonic ΔPMI increase, which may improve local accessibility.

In addition, biofilm viability was further assessed by confocal laser scanning microscopy (CLSM) using a live/dead fluorescence assay (SYTO 9/PI), in which SYTO 9 labels live cells (green fluorescence) and PI labels membrane-compromised/dead cells (red fluorescence)[63]. In agreement with the resazurin assay readout (Fig. 3h and i), the corresponding CLSM z-stacks revealed that BLigNP1-PG provided the most effective inhibition and eradication against *E. coli*, whereas BLigNP1-TA achieved the highest antibiofilm efficacy against *S. aureus* (Fig. 3k).

### 3.4 Biocompatibility and biosafety

**Fig. 4:**
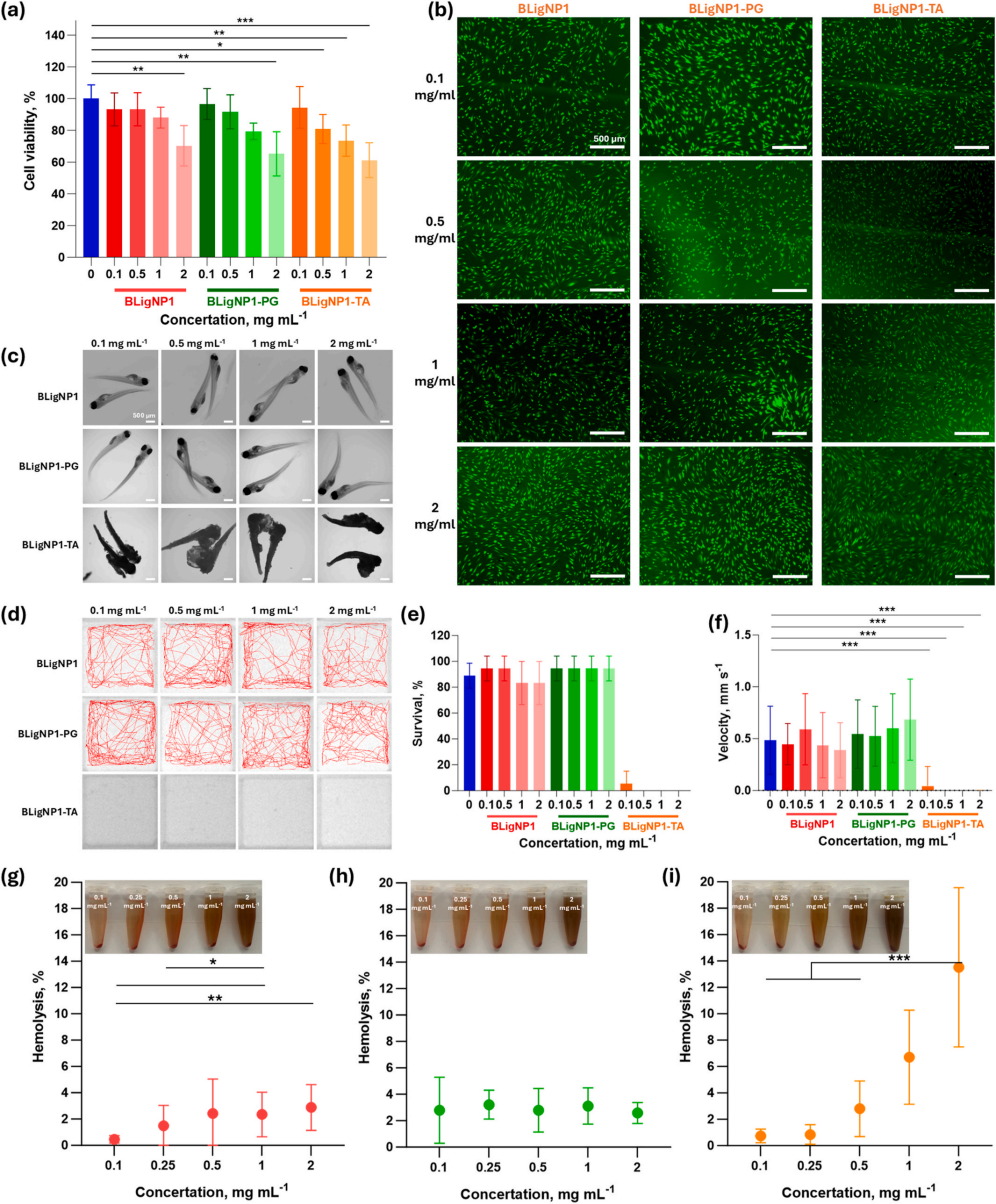
(a) Cell viability of HDFs after treatment with BLigNP1 and BLigNP1-GDs at different concentrations, as determined by the CCK-8 assay. (b) CLSM images of HDFs after treatment with BLigNP1 and BLigNP1-GDs at different concentrations, as assessed by live/dead staining (live cells: green; dead cells: red). Cytotoxicity of BLigNP1 and BLigNP1-GDs at different concentrations in zebrafish, as quantified by changes in their (c) morphology, (d) swimming trajectory, (e) survival, and (f) swimming velocity. Hemolysis assay of (g) BLigNP1, (h) BLigNP1-PG and (i) BLigNP1-TA at different concentrations using sheep erythrocytes. Data are presented as the mean ± SD (n = 3), *p < 0.05, **p < 0.01, ***p < 0.001. Scale bars: 500 μm. (For interpretation of the references to colour in this figure legend, the reader is referred to the Web version of this article.)

Biocompatibility and biosafety are critical for the clinical translation of antibacterial agents, because many membrane-active cationic amphiphiles can cause off-target toxicity by engaging in strong electrostatic and hydrophobic interactions with host cell membranes (phospholipids) and proteins, leading to cytotoxicity and tissue irritation [13]. Accordingly, we first evaluated *in vitro* cytocompatibility using human dermal fibroblasts (HDFs). The Cell Counting Kit-8 (CCK-8) assay showed that cell viability for all treatment groups remained above 75% at 1 mg mL^−1^ (Fig. 4a), whereas colony formation of both *E. coli* and *S. aureus* on solid agar plates was largely absent at the same concentration (Fig. 2i). In accordance with ISO 10993-5, such cell viability levels are generally regarded as indicative of acceptable biocompatibility and suggest minimal cytotoxic effects under the tested conditions. However, all tested polyphenolic NPs induced a marked reduction in cell viability when the concentration reaches 2 mg mL^−1^, indicating a concentration-dependent onset of cytotoxicity. Intriguingly, live/dead staining only revealed a low proportion of dead cells across all treatment groups at varying concentrations (Fig. 4b and Fig. S16–19). These observations suggest that the polyphenolic NPs primarily reduce metabolic activity and proliferation, rather than inducing widespread acute cell death. To further evaluate biocompatibility, hemolysis assays were performed to assess NP-induced hemolysis in sheep erythrocytes. As shown in Fig. 4g–i, BLigNP1 and BLigNP1-PG exhibited hemolysis levels below 5% across the tested concentration range (0.1–2 mg mL^−1^), suggesting acceptable hemocompatibility for prospective applications. However, BLigNP1-TA maintained hemolysis below 5% at 0.1–0.5 mg mL^−1^, but hemolysis increased to ∼6% at 1 mg mL^−1^ and ∼10% at 2 mg mL^−1^, indicating potential hemocompatibility concerns at higher concentrations and warranting caution for applications involving direct blood contact.

Furthermore, an *in vivo* zebrafish embryo acute toxicity assay was conducted to assess potential developmental and systemic toxicity. As exposure routes (*e.g.*, immersion or microinjection) have been shown to significantly affect internal dose, biodistribution, and toxicity outcomes [[64], [65], [66]], we first used microinjection to bypass the chorion and other diffusion barriers and to achieve precise intraembryonic delivery of the polyphenolic NPs. The results showed that, even at a high injection volume (*e.g.*, 23 nL), BLigNP1, BLigNP1-PG and BLigNP1-TA did not cause any apparent changes in morphology (Fig. S20), survival (Fig. S21a), or locomotor behaviour (distance moved, Fig. S21b; swimming velocity, Fig. S21c). Subsequently, immersion exposure to polyphenolic NPs at varying concentrations was initiated at 2 days post-fertilization (2 dpf) to minimize background mortality during early development and to assess toxicity in later-stage embryos, following a procedure previously described in the literature [67,68]. No significant morphological alterations were observed in zebrafish larvae after 48 h of immersion exposure to BLigNP1 or BLigNP1-PG across 0.1–2 mg mL^−1^. However, larvae exposed to BLigNP1-TA displayed pronounced morphological abnormalities, including widespread tissue darkening, and an almost complete loss of recognizable larval structures, indicating severe acute toxicity (Fig. 4c). Consistent with these observations, exposure to BLigNP1-TA across 0.1–2 mg mL^−1^ resulted in decreased larval survival (Fig. 4e). Larval locomotor behaviour was further quantified by tracking swimming velocity (Fig. 4f) and distance moved (Fig. S22). Zebrafish larvae treated with BLigNP1 and BLigNP1-PG exhibited locomotor parameters comparable to negative control larvae (no polyphenolic NPs addition), indicating no detectable behavioural toxicity, whereas BLigNP1-TA exposure resulted in near-complete larval mortality and consequently an almost complete absence of swimming activity (Fig. 4d and f). To further characterize the concentration-dependent toxicity of BLigNP1-TA, additional immersion experiments were performed at lower concentrations. Larval survival remained relatively high at 0.005 mg mL^−1^, reaching approximately 83.3%, but decreased markedly to approximately 27.8% at 0.025 mg mL^−1^ and further declined to approximately 22.2% at 0.05 mg mL^−1^ (Fig. S23). These results demonstrate a clear concentration-dependent toxic response and indicate that a marked decline in survival occurred within the concentration interval of 0.005–0.025 mg mL^−1^ under the tested immersion conditions. However, because the concentrations surrounding this transition were relatively widely spaced and survival was the principal endpoint evaluated, the present data do not permit precise determination of a safety threshold and toxicity window. Therefore, 0.005 mg mL^−1^ should be regarded as a better-tolerated concentration under the tested conditions rather than as an established safe concentration. The pronounced toxicity of BLigNP1-TA may be related to the high surface density of pyrogallol groups in the tannic acid corona, which can promote strong interactions with biological interfaces and context-dependent redox activity [69]. However, the specific mechanisms responsible for the observed zebrafish toxicity were not directly investigated in the present study, and therefore contributions from oxidative stress, epithelial damage, protein interactions, or other interfacial effects cannot yet be distinguished. Consistent with this interpretation, high concentrations of free tannins extracted from eucalyptus leaves have been reported to cause acute toxicity in zebrafish, potentially through strong protein interactions and impairment of blood oxygen-carrying capacity [70].

Overall, these findings indicate that the biocompatibility and biosafety effects of polyphenolic NPs are strongly dependent on both the exposure route and the target tissue. For BLigNP1-TA in particular, no evident adverse effects were detected following intraembryonic microinjection at the tested dose, whereas continuous immersion exposure caused pronounced morphological abnormalities and concentration-dependent larval mortality. This divergence may arise from exposure-route-dependent differences in nanoparticle distribution, local concentration, biological-barrier interactions, and effective internal dose in zebrafish [71]. Differences between the zebrafish and mammalian cell-based assays may additionally be influenced by protein adsorption in serum-containing media, which can alter nanoparticle surface properties and biological interactions [72]. These findings indicate that applications involving prolonged or uncontrolled exposure of epithelial surfaces to BLigNP1-TA would require particular caution. Additional formulation optimization and systematic dose-response studies are needed before a suitable exposure route or application-specific safety range can be established. By comparison, BLigNP1-PG showed better tolerability than BLigNP1-TA under both the tested microinjection and immersion exposure condition.

### 3.5 Antibacterial mechanisms of action

**Fig. 5:**
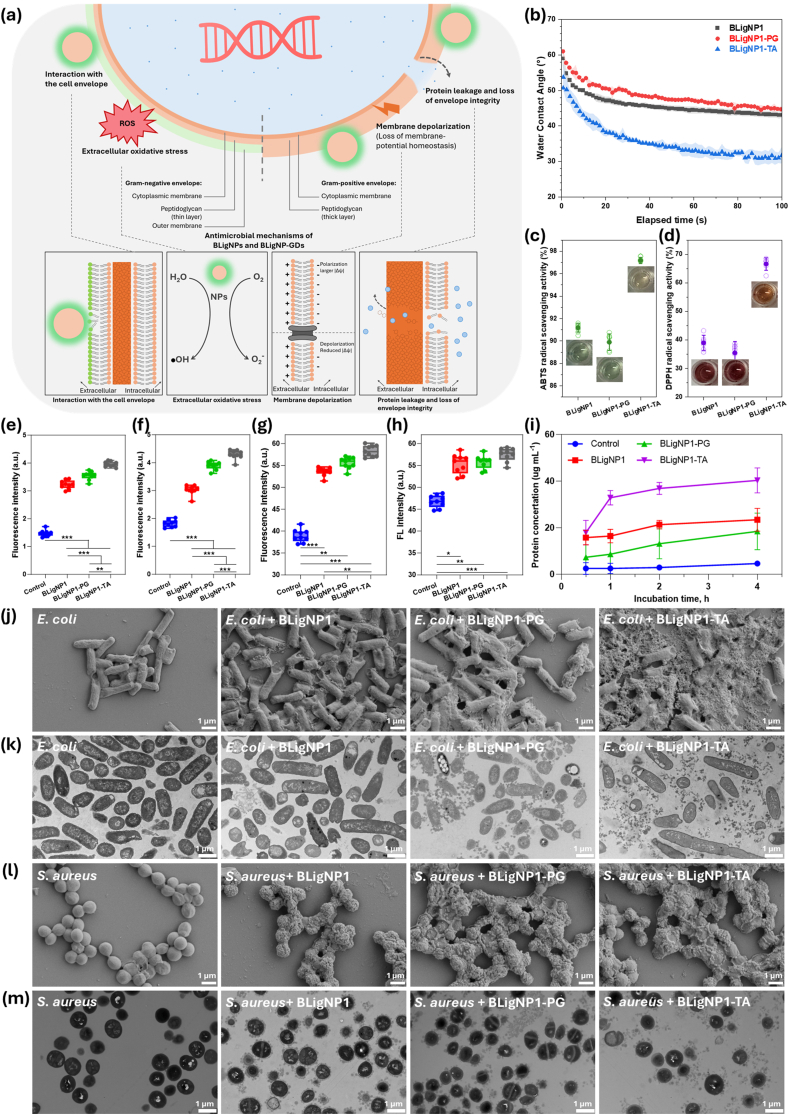
(a) Schematic illustration of the antibacterial mechanism of polyphenolic NPs. (b) Water contact angle as a function of time for BLigNP1, BLigNP1-PG, and BLigNP1-TA. Radical-scavenging capacity determined by (c) ABTS and (d) DPPH. Extracellular ROS generation in (e) *E. coli* and (f) *S. aureus* upon exposure to BLigNP1, BLigNP1-PG, and BLigNP1-TA, measured by the DCFH fluorescence probe. Nanoparticle-induced membrane depolarization in (g) *E. coli* and (h) *S. aureus*, quantified using the voltage-sensitive DiBAC_4_(3) fluorescence probe. (i) Protein leakage from *E. coli* after incubation with BLigNP1, BLigNP1-PG, and BLigNP1-TA. (j) SEM and (k) TEM images of *E. coli* with and without polyphenolic NPs treatment. (l) SEM and (m) TEM images of *S. aureus* with and without polyphenolic NPs treatment. Data are presented as the mean ± SD (n = 3), *p < 0.05, **p < 0.01, ***p < 0.001.

The proposed formulation- and strain-dependent antibacterial mechanism of all-polyphenolic NPs is illustrated in Fig. 5a. Notably, all BLigNP-GDs displayed similarly negative zeta potential in PBS buffer (0.1M, pH 7.4) (Fig. 1e and h), suggesting that the observed differences in antibacterial activity are unlikely to arise primarily from electrostatic interactions. Instead, gallate functionalization introduced formulation-dependent differences in interfacial properties, including relative wettability, surface phenolic hydroxyl density, and the capacity for multivalent interactions with bacterial envelope components and EPS matrix within biofilms. These interfacial differences were supported by the time-dependent water contact angles of the nanoparticle coatings and the surface-tension measurements of the nanoparticle suspensions (Fig. 5b and Fig. S28). Consistent with these differences, BLigNP1-PG exhibited greater activity against *E. coli*, whereas BLigNP1-TA was more effective against *S. aureus* in both planktonic and biofilm assays (Fig. 2g, h and Fig. 3d–**i**).

For *E. coli*, the relatively balanced interfacial properties of BLigNP1-PG may facilitate close association with lipid-rich regions of the bacterial envelope and thereby perturb membrane organization, consistent with previous reports that an optimal degree of hydrophobicity can enhance antibacterial activity against *E. coli* [15,73]. TEM images showed a relatively continuous electron-dense nanoparticle layer closely associated with the *E. coli* envelope following BLigNP1-PG treatment (Fig. 5k). By contrast, BLigNP1-TA appeared predominantly as discrete pericellular deposits and aggregates, which were frequently separated from the cell surface by an electron-lucent region (Fig. 5m). SEM further revealed extensive flocculent and particulate material surrounding BLigNP1-TA-treated cells and promoting bacterial aggregation (Fig. 5j). These observations indicate that BLigNP1-PG exhibits closer envelope association, whereas BLigNP1-TA predominantly undergoes pericellular deposition and aggregation under the examined conditions. The two formulations also produced different functional effects on the *E. coli* envelope. BLigNP1-PG induced membrane depolarization and a measurable increase in extracellular protein concentration, although the extent of protein leakage remained substantially lower than that was induced by BLigNP1-TA (Fig. 5g and i). These findings are consistent with functional membrane perturbation accompanied by comparatively limited macromolecular leakage. By contrast, BLigNP1-TA induced more pronounced membrane depolarization and the greatest extracellular protein leakage, supporting more extensive loss of envelope integrity. Thus, BLigNP1-PG and BLigNP1-TA appear to exert antibacterial effects against *E. coli* through distinct but partially overlapping membrane-associated pathways. BLigNP1-PG primarily promotes close envelope association and disruption of membrane-potential homeostasis with comparatively limited loss of macromolecular integrity, suggesting a predominantly non-lytic mode of action. Similar non-lytic antibacterial effects have been reported for nanomaterials that disrupt membrane potential and impair energy-dependent cellular functions while largely preserving overall envelope integrity [74,75]. BLigNP1-TA, in contrast, induces more extensive envelope permeabilization and leakage of intracellular proteins.

For *S. aureus*, SEM and TEM showed pronounced cell–particle association, bacterial aggregation, and pericellular nanoparticle accumulation following treatment with all three formulations (Fig. 5l and m). All three formulations also induced significant membrane depolarization relative to the untreated control, with no significant differences among the nanoparticle treatment groups (Fig. 5h). These results indicate that association with the Gram-positive cell envelope and disruption of membrane-potential homeostasis are common components of their activity against *S. aureus*. The stronger antibacterial effect of BLigNP1-TA may be associated with its pyrogallol-rich and multivalent surface chemistry, which may enhance interactions with peptidoglycan and envelope-associated proteins. However, the precise molecular interactions responsible for this enhanced activity were not directly determined.

The BLigNP-GDs also exhibited context-dependent redox behavior. BLigNP1-TA displayed the strongest radical-scavenging capacity in the ABTS (Fig. 5c) and DPPH assays (Fig. 5d), consistent with the high density and accessibility of redox-active pyrogallol groups on its surface. These assays evaluate electron- or hydrogen-donating activity toward stable synthetic radicals under cell-free conditions and therefore do not directly predict the net redox behavior of the nanoparticles at the bacteria–nanoparticle interface. In cell-free DCFH measurements performed in physiological buffer, the nanoparticle suspensions produced only a minimal increase in DCF fluorescence (Fig. S31a). In the presence of bacteria, however, extracellular DCF fluorescence increased in a formulation-dependent manner (Fig. 5e and f), whereas no corresponding increase in intracellular DCF fluorescence was detected (Fig. S31c and Fig. S31d). These findings suggest that extracellular ROS are likely generated at NP surfaces and quenched near the bacterial envelope, consistent with reports that surface-generated extracellular ROS contribute to antibacterial activity [76,77]. Nevertheless, because the DCFH-based assay is not specific to individual ROS species and no ROS-scavenger rescue experiments were performed, the observed increase in fluorescence is not sufficient to establish ROS as the primary cause of bacterial killing. Localized extracellular oxidative processes are therefore considered a possible contributor alongside nanoparticle-envelope association, membrane depolarization, and formulation-dependent envelope permeabilization.

## 4 Conclusion

We have demonstrated that the gallate-oriented surface engineering of the corona composition of lignin NPs strengthens the intrinsic antibacterial properties of BLigNP-GDs, an all-polyphenolic NPs platform. The concentration-dependent antibacterial activity of BLigNP-GDs against *E. coli* and *S. aureus* is modulated by their engineered corona composition and resulting surface chemistry. As hydrophobicity increases via PG corona engineering, BLigNP-PG exhibited the highest antibacterial activity against *E. coli* (98.5% CFU reduction at 1 mg mL^−1^), owing to enhanced interactions between the alkyl segments and lipid-rich regions of the outer membrane of Gram-negative bacteria, thereby facilitating membrane insertion and increasing membrane permeability. By spatially grafting of redox-active pyrogallol moieties within a TA-engineered corona, BLigNP-TA was less effective against *E. coli* but showed pronounced inhibition activity against *S. aureus* (98.4% CFU reduction at 1 mg mL^−1^). Rich hydroxyls in polyphenols and high density of pyrogallol moieties in the corona, synergistically promote multivalent noncovalent interactions with the peptidoglycan-rich cell wall of Gram-positive bacteria. Moreover, PG- and TA-engineered corona on the lignin core effectively enhanced NP interactions with biofilm-relevant interfaces and promoting NP penetration and transport within biofilms. Importantly, BLigNP-PG exhibited favorable biocompatibility and biosafety, showing minimal cytotoxicity to mammalian cells and low toxicity in a zebrafish model. Systematic investigations reveal that all-polyphenolic NPs exert antibacterial activity through a multimodal mechanism involving extracellular ROS generation, an increase in cell envelope permeability, and leakage of intracellular contents.

## CRediT authorship contribution statement

**Zonghong Lu:** Data curation, Formal analysis, Investigation, Methodology, Validation, Visualization, Writing – original draft, Writing – review & editing. **Shujun Liang:** Data curation, Investigation, Writing – review & editing. **Depeendra Yadav:** Data curation, Writing – review & editing. **Hao Zhang:** Data curation, Writing – review & editing. **Jessica M. Rosenholm:** Resources, Writing – review & editing. **Chunlin Xu:** Resources, Writing – review & editing. **Tapani Viitala:** Formal analysis, Investigation, Methodology, Validation, Writing – review & editing. **Xiaoju Wang:** Conceptualization, Funding acquisition, Project administration, Resources, Supervision, Validation, Writing – review & editing.

## Declaration of competing interest

The authors declare that they have no known competing financial interests or personal relationships that could have appeared to influence the work reported in this paper.

## Data Availability

Data will be made available on request.

## Appendix A Supplementary data

Materials and Chemicals; Instrumental setups and detailed experimental procedures for BLigNP-GD characterization; Calibration curve for protein quantification (Fig. S1); SEM images of BLigNP-GD coated aluminum surface (Fig. S2); Water contact angle for the aluminum container (Fig. S3); Average of real‐time MP‐SPR responses during biofilm formation (Fig. S4); TEM images of BLigNPs (Fig. S5); ^11^B NMR of BLigNPs (Fig. S6); Hydrodynamic size distribution and PDI of BLigNPs and BLigNP-GDs (Fig. S7, 8 and 10); Growth curves of *S. aureus* (Fig. S9); Growth inhibition of *E. coli* and *S. aureus* (Fig. S11); Digital image of colony formation on agar-plate (Fig. S12); The changes of hydrodynamic size and zeta potential over time (Fig. S13); Antibacterial durability of BLigNP1 and BLigNP-GDs (Fig. S14); Average of real‐time MP‐SPR responses at 785 nm (Fig. S15); Live/dead staining of untreated and treated HDFs (Fig. S16–S19); Representative images of zebrafish embryos (Fig. S20); Locomotor effects of BLigNP1 and BLigNP1-GDs (Fig. S21); Total distance moved by zebrafish embryo (Fig. S22); Survival of zebrafish embryos (Fig. S23); Effects of the positive control on zebrafish embryos (Fig. S24–S25); Digital image of hemolysis controls (Fig. S26–S27); Surface tension of BLigNP1 and BLigNP1-GDs dispersions (Fig. S28); TEM images of untreated and treated *E. coli* and *S. aureus* (Fig. S29–S30); ROS generation (Fig. S31); Protein leakage of *S. aureus* (Fig. S32);[31]P NMR spectra of WSAL and boronated lignin (Fig. S33); Molar mass and dispersity (Table S1) and the amount of OH group (Table S2) of lignin powder and BLigNP.
