# Supplementary material for: Gallate-oriented surface engineering of lignin nanoparticles for enhanced antibacterial activity
Source: Mater Today Bio. 2026 Jul 31;40:103517. doi: 10.1016/j.mtbio.2026.103517 (PMC13473958; doi:10.1016/j.mtbio.2026.103517)
Supplement: Supplementary file 1 [file mmc1.pdf]

Supplementary Information

Gallate-oriented Surface Engineering of Lignin Nanoparticles for Enhanced Antibacterial Activity

Zonghong Lu[a], Shujun Liang[a,b], Depeendra Yadav[b], Hao Zhang[a], Jessica M. Rosenholm[b], Chunlin Xu[a], Tapani Viitala[b], Xiaoju Wang[a,b]**

[a] Laboratory of Natural Materials Technology, Department of Engineering and Information Technology, Åbo Akademi University, Henrikinkatu 2, Turku FI-20500, Finland

[b] Pharmaceutical Sciences Laboratory, Department of Natural and Health Sciences, Åbo Akademi University, Tykistökatu 6A, Turku FI-20520, Finland

*Corresponding authors' email addresses: tapani.viitala@abo.fi (T. Viitala) and xwang@abo.fi (X. Wang)

Characterizations

Particle size and zeta potential. Hydrodynamic particle size distributions and zeta potentials of the NPs were measured using a Zetasizer Nano (Malvern Panalytical, UK) at room temperature (25 °C). The particle refractive index was set to 1.595 in the instrument software.

Fourier-transform infrared spectroscopy (FTIR) analysis. FTIR spectra were recorded using a Nicolet iS50 FTIR spectrometer (Thermo Fisher Scientific, USA) equipped with an attenuated total reflectance (ATR) accessory. Spectra were collected from 4000 to 400 cm^{-1} at a resolution of 4 cm^{-1}, averaging 64 scans per sample.

Size-exclusion chromatography (SEC) analyses. The molar mass of BLigNPs was determined by a SEC equipped with a multi-angle light scattering (MALS) and a differential refractive index (RI) detector (Agilent + Wyatt, USA), following our previous protocol [1]. Separation was achieved using a Jordi Gel GBR Mixed-Bed column (10 × 250 mm, I.D. × L) with a Jordi Gel Glucose Mixed-Bed guard column (10 × 50 mm, I.D. × L). The mobile phase was HPLC-grade DMSO containing 0.05 M lithium bromide (LiBr), operated at 60 °C with a flow rate of 0.5 mL min^{-1} for 50 min. Samples were injected at a concentration of 5 mg/mL with an injection volume of 100 µL. Data were processed using ASTRA software (v7.3.2) with a refractive index increment (dn/dc) of 0.15 mL g^{-1}.

Quantitative ^{31}P NMR analysis. All NMR experiments were performed on a Bruker AVANCE III 500 MHz spectrometer (Bruker, Karlsruhe, Germany) equipped with a BBO probe. Hydroxyl group contents were determined by quantitative ^{31}P NMR, following our previously established method [2]. In short, 20 mg of the pre-dried sample was dissolved in 400 µL of anhydrous pyridine and deuterated chloroform ($CDCl_3$) mixture (1.6:1, v/v). Next, 100 µL of endo-N-hydroxy-5-norbornene-2,3-dicarboximide (internal standard, 0.12 mmol mL^{-1} in pyridine/CDCl) 50 µL of chromium(III) acetylacetonate ($Cr(acac)_3$) (relaxation reagent, 11.4 mg/mL in pyridine/CDCl) were added, giving a $Cr(acac)_3$ final concentration of 0.002 M. The mixture was then phosphitylated with 80 µL of 2-chloro-4,4,5,5-tetramethyl-1,3,2-dioxaphospholane (Cl-TMDP) for 30 mins before transfer to an NMR tube for analysis. Quantitative ^{31}P NMR spectra were acquired with a relaxation delay of 10 s, an acquisition time of 1.63 s, 80 scans, and the temperature maintained at 298 K.

^{11}B NMR for BLigNP dispersions in D_2O. Surface boronate functionalities of BLigNPs were analyzed by ^{11}B NMR (500.13 MHz, 298 K). An aliquot corresponding to 20 mg of BLigNP was collected by centrifugation (MiniSpin microcentrifuge, Eppendorf; 12,000 rpm,

10 min). Then, the supernatant was removed by decantation, and the BLigNP pellet was redispersed in 500 μL of D_2O. ^{11}B NMR spectra were acquried on a Bruker AVANCE III 500 MHz spectrometer (Bruker, Karlsruhe, Germany) equipped with a 5 mm Z-gradient BBO (Broadband Observe) probe using an acquisition time of 1.0 s, a relaxation delay of 1.0 s, and 1000 scans.

Revival of bacteria from frozen stocks. Cryovials were removed from the -80 °C freezer and kept on ice to minimize thawing. A small amount of the frozen glycerol stock was aseptically scraped with a sterile inoculation loop and streaked onto an agar plate. The plates were incubated inverted at 37 °C for 15 h. A single well-isolated colony was then picked and inoculated into LB broth or TS broth, followed by incubation at 37 °C with shaking to obtain an overnight bacterial culture.

***In vitro* antibacterial activity of BLigNP-GDs.** The antibacterial activity of BLigNP-GDs was evaluated using both growth curve analysis and a colony-forming unit (CFU) plate-counting assay. Overnight bacterial culture was diluted in LB broth to a final density of 1×10^6 CFU mL^{-1}. Then, 100 μL of BLigNP-GD suspensions at various concentrations were mixed with 100 μL of bacterial suspension in a 96-wellplate and incubated at 37 °C with shaking. Bacteria grown in LB broth without NPs used as the growth control.

Growth curve was monitored by measuring the optical density at 600 nm (OD_{600}) using a Varioskan Flash Multilabel device (Thermo Scientific, USA). All presented OD values were obtained after subtracting the background OD of the nanoparticles themselve. For the plate-counting, the treated bacterial suspension was diluted 10^5-fold or 10^6-fold in PBS and an aliquot (50 μL) of the diluted suspensions was spread on agar culture plates. After 20 h incubation at 37 °C, colonies on agar plates were counted.

***In vitro* antibiofilm assay.** To evaluate biofilm inhibition, *S. aureus* and *E. coli* were exposed to polyphenolic NPs during the initial association and biofilm development stage. Briefly, 50 μL of an overnight bacterial culture (1×10^7 CFU mL^{-1}) and 50 μL of polyphenolic NPs dispersion in TS broth (2 mg mL^{-1}) were added to each well of a 96-well plate and incubated at 37 °C for 24 h. After incubation, the planktonic phase was removed and gently washed with PBS. The metabolic activity was then assessed by adding resazurin (final concentration 0.1%) and measuring fluorescence using a Varioskan Flash Multilabel device (Thermo Scientific, USA) with excitation at 530 nm and emission at 590 nm.

To evaluate biofilm eradication, mature biofilms were first established by seeding 100 μL of an overnight bacterial culture (1×10^7 CFU mL^{-1}) in TS broth in a 96-well plate and incubating at 37 °C for 24 h. After biofilm formation, the planktonic phase was removed and gently washed with PBS. Then, fresh TS broth (100 μL) and polyphenolic NPs dispersion in TS broth (100 μL, 2 mg mL^{-1}) were then added, and the plates were incubated for an additional 24 h at 37 °C. Biofilm metabolic activity after treatment was quantified using the resazurin assay as described in biofilm inhibition section.

Analysis of morphology of bacteria. Confocal laser scanning microscopy (CLSM) imaging of biofilms: Biofilms were grown in glass-bottom 96-well plates following the same procedures used for the biofilm inhibition and eradication assays. After removing planktonic bacteria and washing with PBS, biofilms were fixed with 4% paraformaldehyde for 20 min and washed three times with PBS (10 min per wash). They were then stained with the LIVE/DEAD™ BacLight bacterial viability kit (SYTO 9, 6.7 μM; propidium iodide, 40 μM) for 20 min in the dark. After staining, the staining solution was removed, and the biofilms were rinsed three times with PBS to remove excess dye. Fluorescence images and 3D biofilm structures were acquired using a CLSM system (Zeiss LSM 880 with AiryScan).

Scanning Electron Microscopy (SEM) imaging of bacteria after polyphenolic NPs treatment: An overnight bacterial culture (500 μL, 1×10^7 CFU mL^{-1}) was mixed with 500 μL of a polyphenolic NPs dispersion (2 mg mL^{-1}) in TS broth and incubated at 37 °C for 15 h. Cells were collected by centrifugation (5000 × g, 5 min), the supernatant was discarded, and the pellet was washed with PBS. Then samples were fixed in 2.5% (v/v) glutaraldehyde at room temperature for 1 h, rinsed with PBS. The fixed samples were deposited onto a sterile silica wafer and were subsequently dehydrated through a graded ethanol series (50%, 70%, 80%, 90%, 95%, and 100% v/v) for 10 min at each step.

Transmission Electron Microscope (TEM) imaging of bacteria after NPs treatment: Following fixation and dehydration as described above in SEM sample preparation, the samples were incubated in 100% propylene oxide for 15 min, infiltrated and embedded in epoxy resin, and polymerized at 60 °C overnight. Ultrathin sections (50–100 nm) were cut using an ultramicrotome and collected on copper grids. The sections were stained with 1% (w/v) uranyl acetate for 1 min at room temperature. After blotting dry with filter paper, the grids were analysed by TEM.

Monitoring NPs-bacterial biofilm interactions using Multi-Parametric Surface Plasmon Resonance (MP-SPR). MP-SPR measurements were carried out on a BioNavis MP-SPR 200 instrument (BioNavis) equipped with gold-coated sensor, following the procedure described previously with minor modifications [3]. Overnight bacterial cultures (~1 × 10^7 CFU) were seeded *in situ* on gold-coated sensor surfaces at 37 °C by injecting the cell suspension through four independent flow channels at a constant flow rate of 50 μL min^{-1}. Bacteria were then continuously perfused for 20 h to allow the formation of confluent biofilms on the sensor surface prior to NPs exposure. Biofilm maturation was confirmed by the stabilization of the SPR signals, as the ΔPAP response approached a plateau and the ΔPMI signal stabilized toward the end of the perfusion period, indicating that the biofilm layer within the evanescent-field region had reached a quasi-steady state (**Fig. S1**).

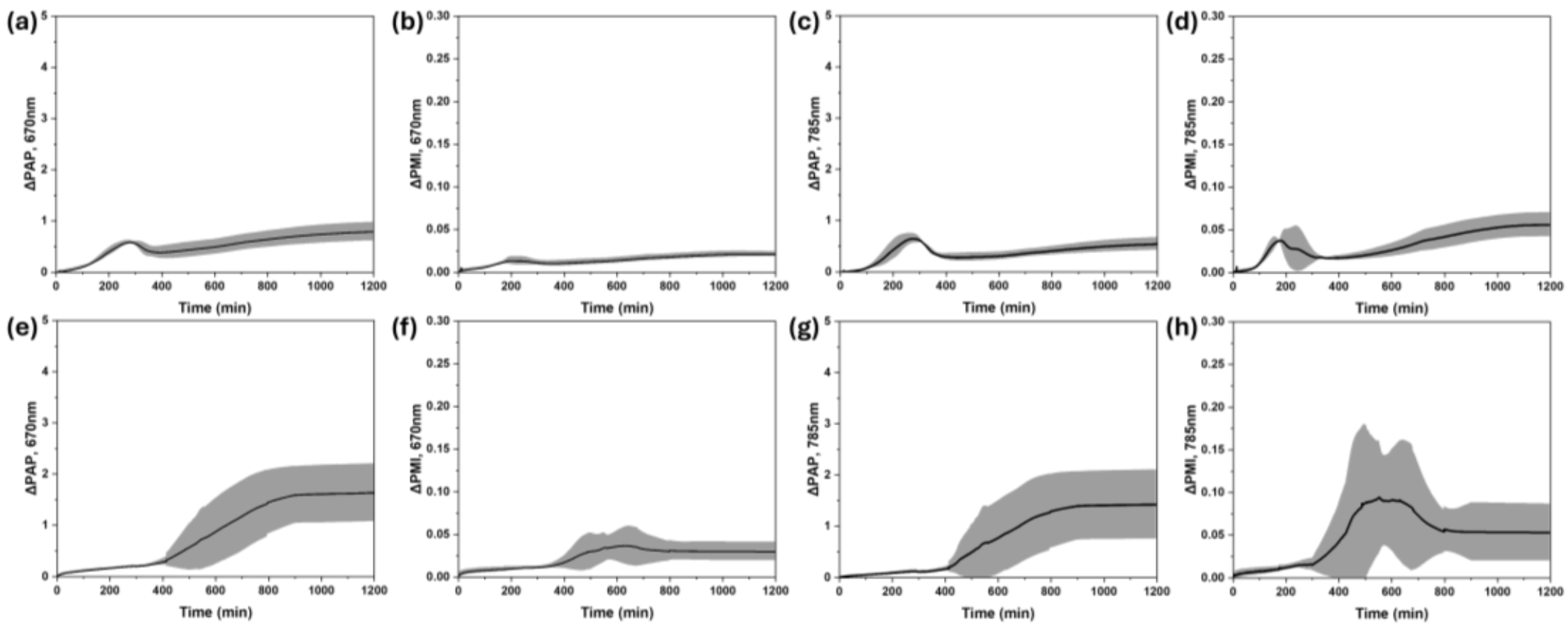

Fig. S1. Average of real-time MP-SPR responses during *E. coli* bacterial biofilm formation (0–1200 min): (a) ΔPAP at 670 nm, (b) ΔPMI at 670 nm, (c) ΔPAP at 785 nm, and (d) ΔPMI at 785 nm. Average of real-time MP-SPR responses during *S. aureus* bacterial biofilm formation (0–1200 min): (e) ΔPAP at 670 nm, (f) ΔPMI at 670 nm, (g) ΔPAP at 785 nm, and (h) ΔPMI at 785 nm.

After biofilm establishment, a new measurement sequence was initiated by perfusing fresh TSB for 10 min to flush the flow channels and remove loosely attached (reversibly adherent) cells. Polyphenolic NPs dispersions (100 μg mL^{-1}) were then introduced and continuously perfused for an additional 20 h to monitor NP–biofilm interactions in real time.

Cytotoxicity assessments of human dermal fibroblasts (HDFs). HDFs were used as a representative mammalian host cell line and cultured in DMEM supplemented with 10% FBS and 1% pen–strep (hereafter referred to as complete DMEM) in a humidified incubator at 37 °C and 5% CO_2. Unless otherwise stated, complete DMEM was used throughout. HDFs

were seeded in 96-well plates at 10000 cells per well and allowed to adhere for 48 h. The medium was then replaced with fresh complete DMEM containing polyphenolic NPs at various concentrations (0.1–2 mg mL^{-1}) and incubated for an additional 24 h. After exposure, the NPs-containing medium was removed, and the cells were allowed to recover for 24 h in fresh complete DMEM.

Cell viability was first evaluated using the CCK-8 assay. Briefly, the medium was removed and 100 µL of CCK-8 working solution (10% v/v CCK-8 reagent in complete DMEM) was added to each well, followed by incubation at 37 °C for 2 h. Absorbance was recorded at 450 nm using a Varioskan Flash multimode plate reader (Thermo Scientific, USA). Cell viability (%) was calculated according to Equation S1:

$$Cell\ viability(\%) = (OD_{sample}\text{-}OD_{blank})/(OD_{control}\text{-}OD_{blank})*100 \quad \text{(S1)}$$

Where, OD_{sample}: absorbance of polyphenolic NPs treated cells; $OD_{control}$: absorbance of untreated control cell; OD_{blank}: absorbance of blank wells (containing only complete DMEM and CCK-8).

Cell viability was further assessed using live/dead (Calcein AM/EthD-III) staining, where live and dead cells fluoresce green and red, respectively. After the 24 h recovery period, the medium was removed and 100 µL of staining solution (0.1125 mM Calcein AM and 2 mM EthD-III in PBS) was added to each well. Samples were incubated for 30 min in the dark, and fluorescence images were acquired using an inverted fluorescence microscope (Zeiss Axio Vert) equipped witha 5×objective.

Hemolysis assay. To evaluate the hemocompatibility of the polyphenolic NPs, a hemolysis assay was performed. Sheep blood was centrifuged at 500 × g for 5 min to pellet red blood cells (RBCs). The RBC pellet was washed with PBS (pH 7.4, 10 × pellet volume) and centrifuged until the supernatant was clear. The washed RBCs were then resuspended in PBS to prepare a 5% (v/v) RBCs suspension. Equal volumes (500 µL each) of the RBC suspension and NPs dispersions were mixed in microcentrifuge tubes. After incubation at 37 °C for 1 h, the samples were centrifuged at 500 × g for 5 min, and the absorbance of the supernatants was recorded at 545 nm using a Varioskan Flash multimode plate reader (Thermo Scientific, USA). Hemolysis (%) was calculated according to Equation S2:

$$Hemolysis\ (\%) = \frac{A_{sample} - A_{negative}}{A_{positive} - A_{negative}} * 100 \quad \text{(S2)}$$

where A_{sample}: is the absorbance of the sample supernatant, $A_{negative}$ is the absorbance of the PBS control, and $A_{positive}$: is the absorbance of the DI water control.

***In vivo* cytotoxicity assessment in a zebrafish embryo model.** Wild-type fertilized zebrafish embryos were collected immediately after spawning and maintained in E3 medium at 28.5 °C in a humidified incubator. Healthy, normally developing embryos were selected at 4–10 h post-fertilization (hpf) and used for either microinjection or immersion exposure. E3 medium served as the negative control, and 3,4–dichloroaniline (4 mg L^{-1}) was used as the positive control. For all treatment and control groups, locomotor behaviour was recorded between 14:00 and 17:00 to minimize circadian influences.

Microinjection: Selected healthy embryos (4–10 hpf) were aligned in grooves of a 1–2% (w/v) agarose injection mold submerged in E3 medium. Injection needles were pulled from borosilicate glass capillaries using a micropipette puller and mounted on a micromanipulator connected to a pneumatic microinjector. Polyphenolic NPs dispersions (1 mg mL^{-1}) were freshly prepared in E3 medium and supplemented with 0.05% (w/v) phenol red as a visual tracer. NPs were microinjected into the yolk sac. The injection volume was calibrated under a stereomicroscope by droplet measurement and set to 2.3, 9.6, or 23 nL per embryo. Injection pressure and injection duration were kept constant for each volume setting. After injection, embryos were gently released from the agarose, rinsed with fresh E3, and incubated at 28.5 °C until evaluation. Survival was assessed at 96 hpf, and locomotor activity (total distance traveled and swimming velocity) was quantified using a DanioVision recording system equipped with EthoVision XT (v11). Embryos were then fixed in 4% paraformaldehyde (PFA) overnight and imaged using a Zeiss AxioZOOM.V16.

Immersion exposure: Selected healthy embryos (4–10 hpf) were cultured in E3 medium at 28.5 °C in a humidified incubator for 48 h. Embryos were then exposed to E3 medium containing polyphenolic NPs at different concentrations and incubated for an additional 48 h (total 96 hpf). After exposure, survival was assessed, and locomotor activity (total distance traveled and swimming velocity) was quantified using a DanioVision recording system equipped with EthoVision XT (v11). Embryos were then fixed in 4% paraformaldehyde (PFA) overnight and imaged using a Zeiss AxioZOOM.V16.

Water contact angle. The water contact angle of polyphenolic NPs was measured by static contact angle measurement (KSV CAM200, KSV Instruments Ltd., Finland) using a sessile drop method. Briefly, 5 mL of NPs suspension (1 mg mL^{-1}) dispersed in Milli-Q water was

dried in an aluminium container at 60 °C overnight to form a NP-coated surface. SEM confirmed high and uniform NPs coverage on the aluminium substrate (**Fig. S2**), ensuring that the water droplet contacted the NPs layer rather than the bare metal. For water contact angle measurements, a 4 µL Milli-Q water droplet was deposited on the surface, and water contact angle were recorded every second for 100 s at 20°C.

Reported water contact angle values represent the mean of three measurements taken at different positions on each sample. To account for any contribution from the substrate, the water contact angle of the bare aluminium container (without polyphenolic NPs coating) was measured separately and used to exclude substrate effects on the lower water contact angle values observed for NPs thin layers (**Fig. S3**).

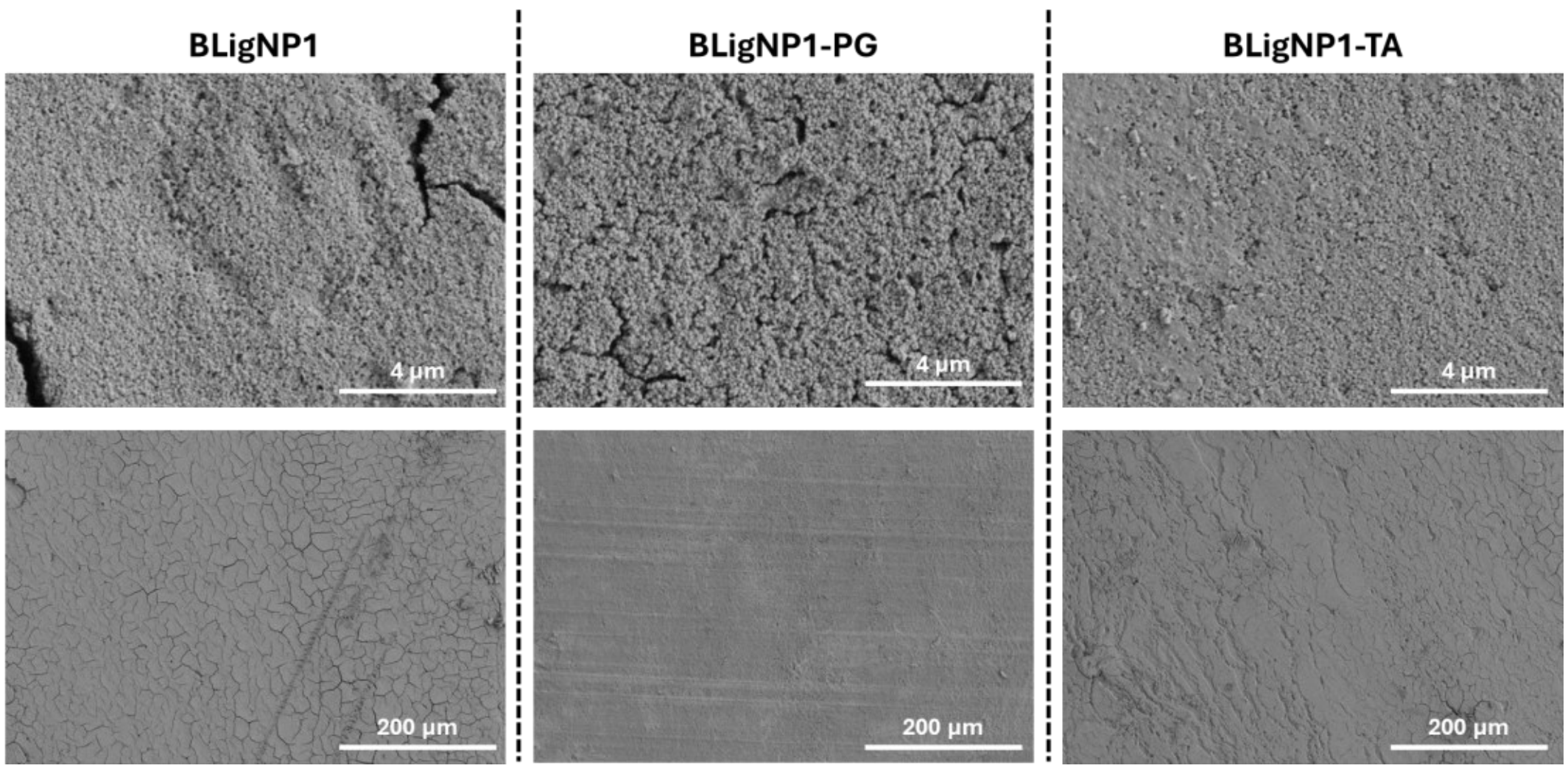

Fig. S2. SEM images of BLigNP1, BLigNP1-PG, and BLigNP1-TA coated aluminum surface for water contact angle measurement.

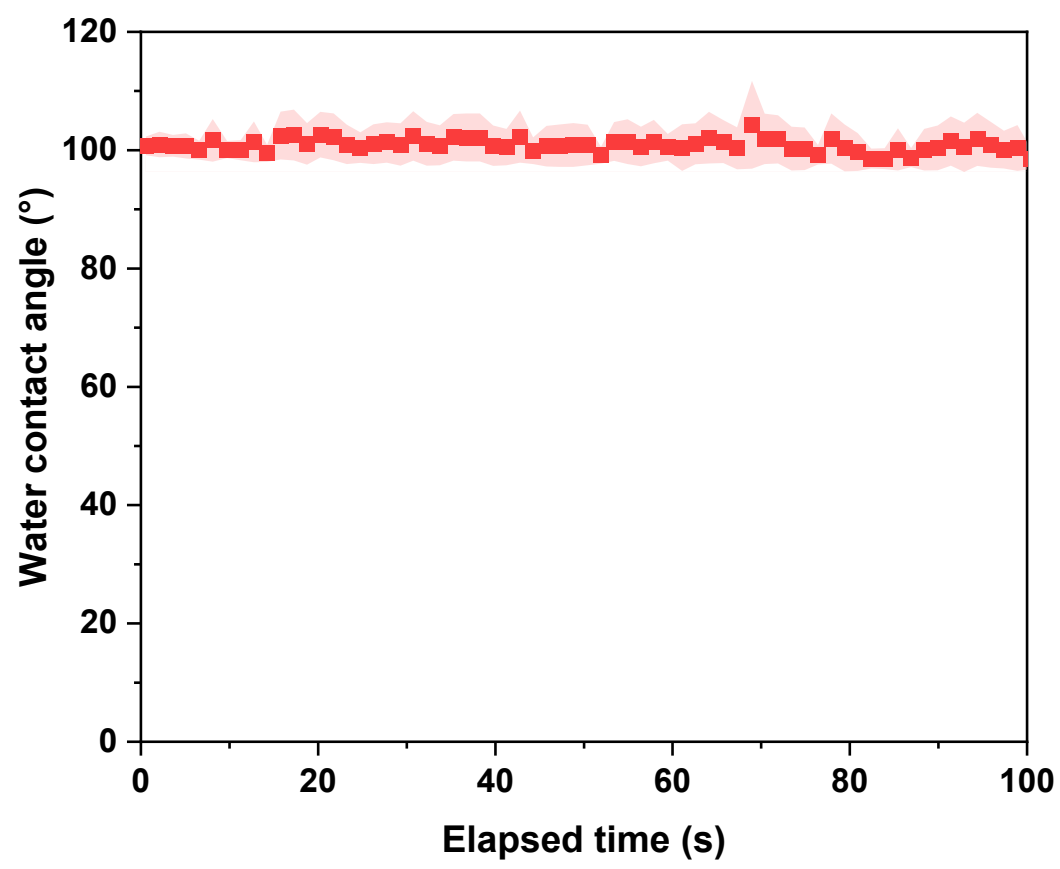

Fig. S3. Water contact angle as a function of time for the aluminum container.

Surface Tension. The air-liquid surface tension of NPs dispersions (10 mg mL^{-1} in PBS) was measured at 25 °C using a force tensiometer (K100, Krüss, Germany) equipped with a Wilhelmy plate.

ABTS radical scavenging assay. The ABTS radical solution was prepared by mixing a 7 mM ABTS stock solution with 2.45 mM potassium persulfate and incubating the mixture in the dark at room temperature for 12 h until the absorbance stabilized. The resulting ABTS radical solution was then diluted with PBS to obtain the ABTS working solution. For the assay, 10 µL of each sample (1 mg mL^{-1}) was added to 190 µL of the ABTS working solution and incubated in the dark at room temperature for 30 min. The percentage of ABTS scavenging activity was calculated based on the absorbance at 734 nm using the following Equation S3:

$$ABTS\ scavenging\ (\%) = \left(A_{control} - A_{sample}\right) A_{control} \quad \text{(S3)}$$

Where, A_{sample}: absorbance of treated samples; $A_{control}$ aborbance of control sample (containing only ABTS).

DPPH radical scavenging assay. DPPH was dissolved in methanol to prepare a working solution (0.1 mg mL^{-1}). Then, 10 µL of each sample (1 mg mL^{-1}) was mixed with 190 µL of the DPPH working solution. After incubation in the dark at room temperature for 30 min, the absorbance at 517 nm was measured using a Varioskan Flash Multilabel device (Thermo Scientific, USA). The percentage of DPPH radical scavenging activity was calculated using Equation S4:

$$DPPH\ scavenging\ (\%) = \left(A_{control} - A_{sample}\right)/A_{control} * 100 \quad \text{(S4)}$$

Where, A_{sample}: absorbance of treated samples; $A_{control}$ aborbance of control sample (containing only DPPH).

ROS Burst detection. Intracellular ROS in bacteria was quantified using the DCFH-DA fluorescence assay. Briefly, 400µL of overnight bacterial culture (~1×10^8 CFU) was incubated with 400 µL of DCFH-DA solution (40 µM) for 30min incubation at 37 °C in the dark. Cells were then collected by centrifugation (5000 × g, 5 min), washed with PBS to remove residual probe, and resuspended in PBS. The probe-loaded bacteria were subsequently exposed to NPs (final concertation 1mg mL^{-1}) and incubated for an additional 30 min at 37 °C in the dark. Fluorescence was recorded using a Varioskan Flash Multilabel device (Thermo Scientific, USA) (λ_ex = 488 nm; λ_em = 525 nm).

Extracellular ROS generation was determined using the non-fluorescent probe 2′,7′-dichlorodihydrofluorescein (DCFH), which can be oxidized to the fluorescent product DCF. Briefly, 50 μL of overnight bacterial culture (~1 × 10^8 CFU) was first mixed with 50 μL of NPs dispersion (2 mg mL^{-1}) and incubated for 30 min at 37 °C. DCFH was freshly prepared by diluting 40 μL of a 10 mM DCFH-DA stock solution with 360 μL of 0.01 M NaOH (5 min on ice, protected from light) to obtain 1 mM DCFH solution, followed by dilution with 9.6 mL PBS to yield 40 μM DCFH. The bacterial and polyphenolic NPs mixtures were then mixed with the 40 μM DCFH solution and incubated for a further 30 min at 37 °C in the dark. Cells were then collected by centrifugation (5000 × g, 5 min, washed with PBS to remove residual probe, and resuspended in PBS. Fluorescence was measured using a Varioskan Flash Multilabel device (Thermo Scientific, USA) (λ_{ex} = 488 nm; λ_{em} = 525 nm).

Bacterial membrane potential assay. The membrane potential of bacteria was measured using a fluorescence-based assay with the voltage-sensitive dye $DiBAC_4(3)$. $DiBAC_4(3)$ exhibits increased fluorescence when cells depolarize (membrane potential becomes less negative) and decreased fluorescence when cells hyperpolarize. Overnight bacterial culture (~1 × 10^8 CFU) were treated with polyphenolic NPs for 4 h at 37 °C. Cells were washed with PBS by centrifugation (5000 × g, 5min) and resuspended in 200 μL of PBS. Subsequently, 200 μL of $DiBAC_4(3)$ solution (20 μM) was added, and the mixture was incubated for 30 min at 37 °C in the dark. After staining, cells were washed with PBS by centrifugation (5000 × g, 5 min) to remove excess dye and resuspended in PBS. The fluorescence intensity was measured by a Varioskan Flash Multilabel device (Thermo Scientific, USA) (λ_{ex} = 490 nm; λ_{em} = 505nm).

Protein leakage assay. Protein leakage from bacteria cells was determined using a Coomassie (Bradford) protein assay kit. A calibration curve was obtained using serial dilutions of BSA, yielding the linear equation $y = 4.504x + 0.1687$ ($R^2 = 0.9947$), where y is absorbance and x is protein concentration (mg mL^{-1}).

Overnight bacterial cultures were collected by centrifugation (5000 × g, 5 min) and washed with PBS. The cells were resuspended in PBS and treated with polyphenolic NPs, followed by incubation at 37 °C for 0, 0.5, 1, 2, or 4 h. After treatment, samples were centrifuged (5000 × g, 5 min) to collect the supernatants. Aliquots of supernatant (50 μL) were mixed with 100 μL of Coomassie reagent and incubated at 37 °C for 5 min, after which absorbance was measured at 592 nm using Varioskan Flash Multilabel device (Thermo Scientific, USA). Protein leakage was calculated as the net increase in extracellular protein relative to the 0 min baseline.

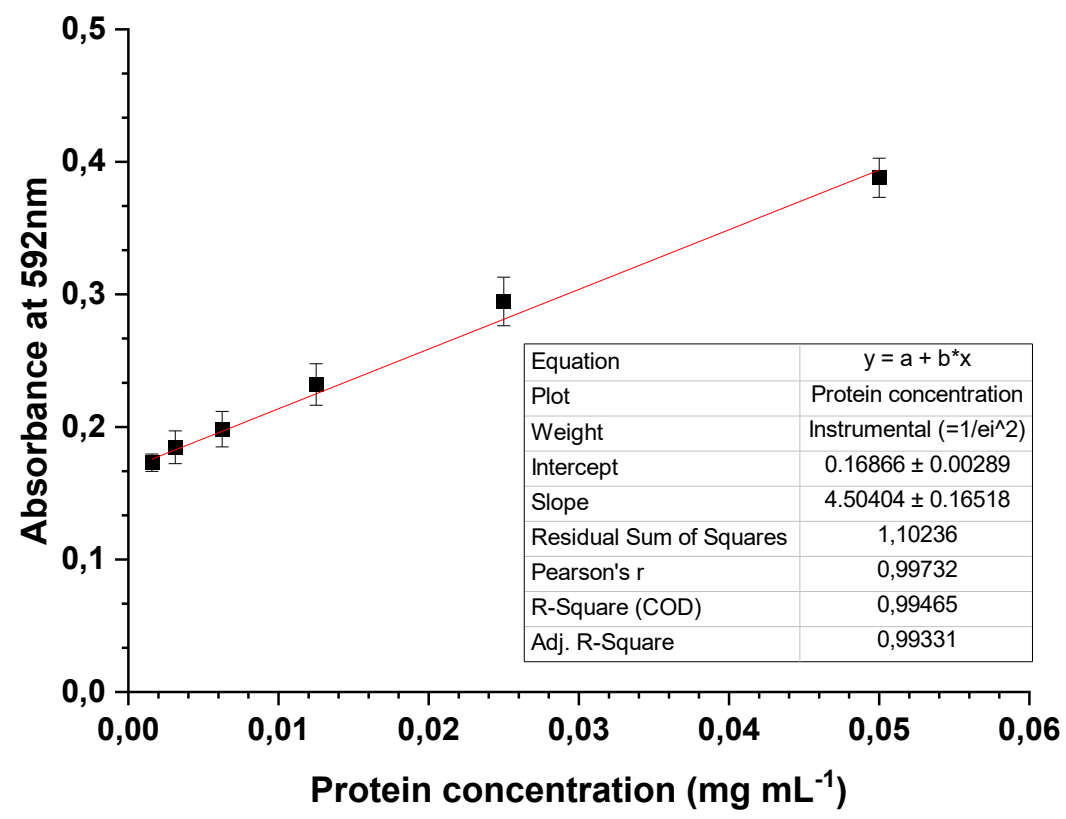

Fig. S4. Calibration curve for protein quantification based on a series dilution of BSA standards.

Statistical analysis. Data processing and statistical analyses were performed using Origin 2022 and GraphPad Prism 10. All results are presented as mean ± standard deviation (SD) from three independent replicates ($n = 3$). Group comparisons were conducted using one-way analysis of variance (ANOVA) at a 95% confidence level. Statistical significance is indicated by asterisks (*$p < 0.05$, **$p < 0.01$, ***$p < 0.001$).

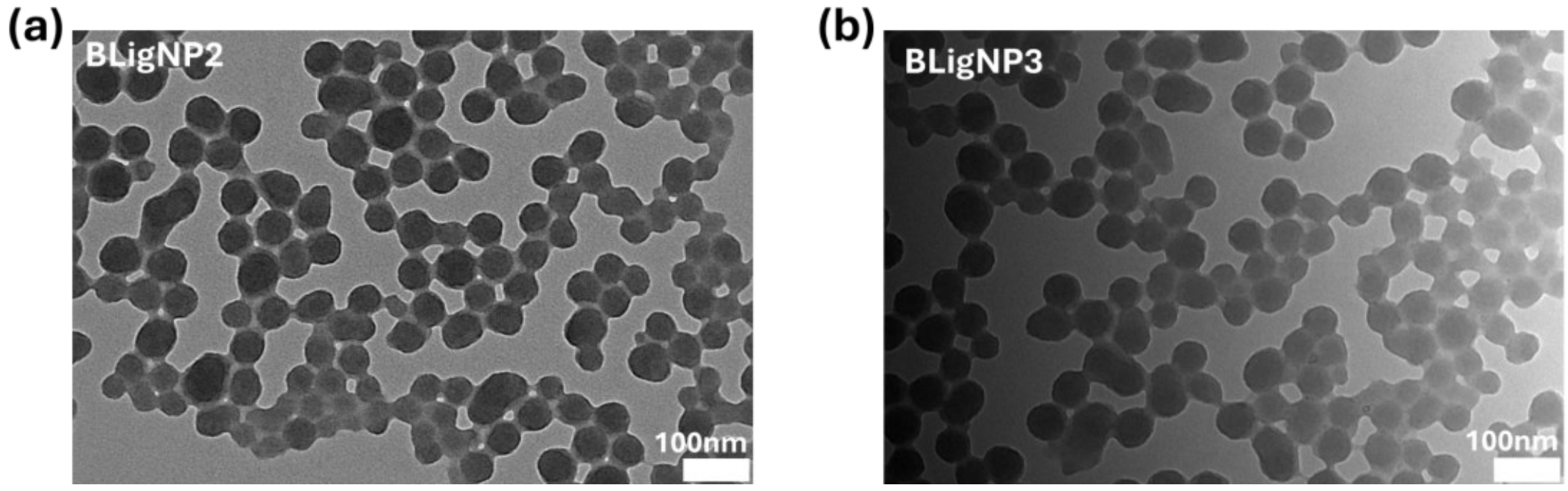

Fig. S5. TEM images of the (a) BLigNP2 and (b) BLigNP3.

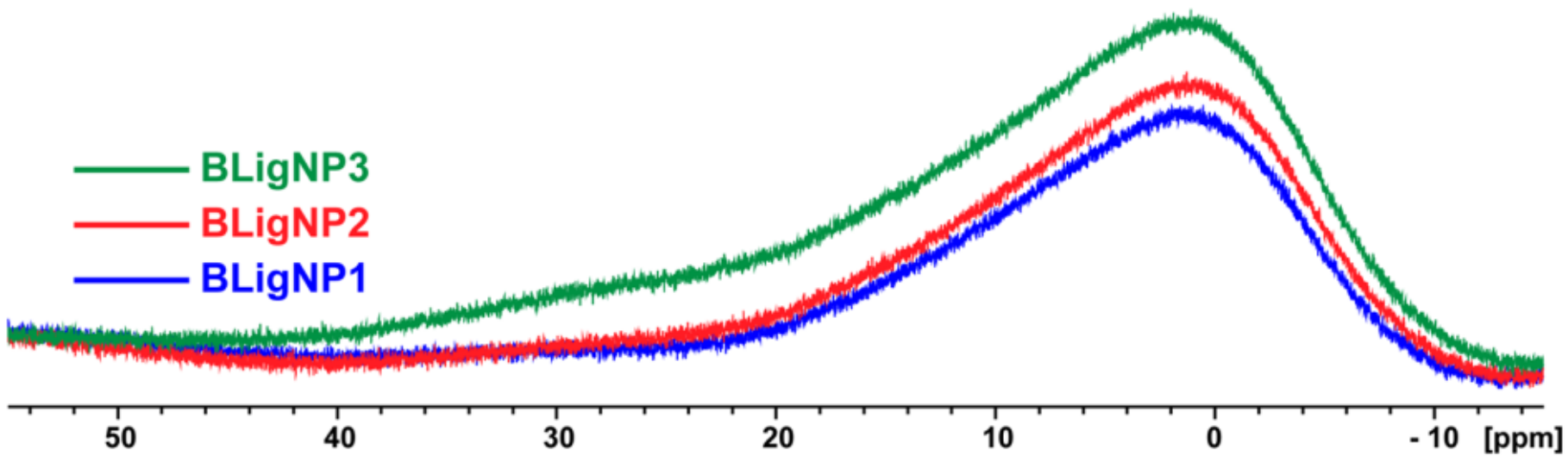

Fig. S6. ^{11}B NMR of D_2O-dispersed BLigNPs.

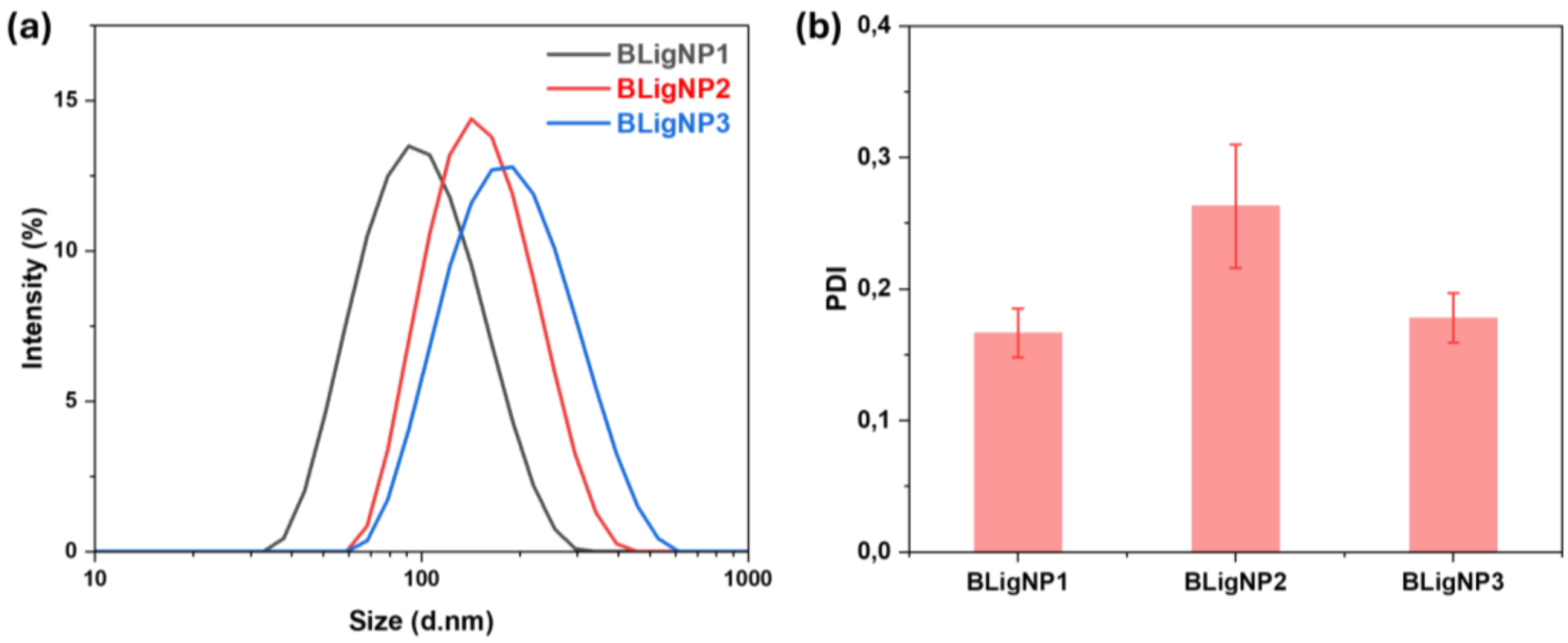

Fig. S7. (a) Hydrodynamic size distribution and (b) polydispersity index (PDI) of BLigNPs measured by DLS.

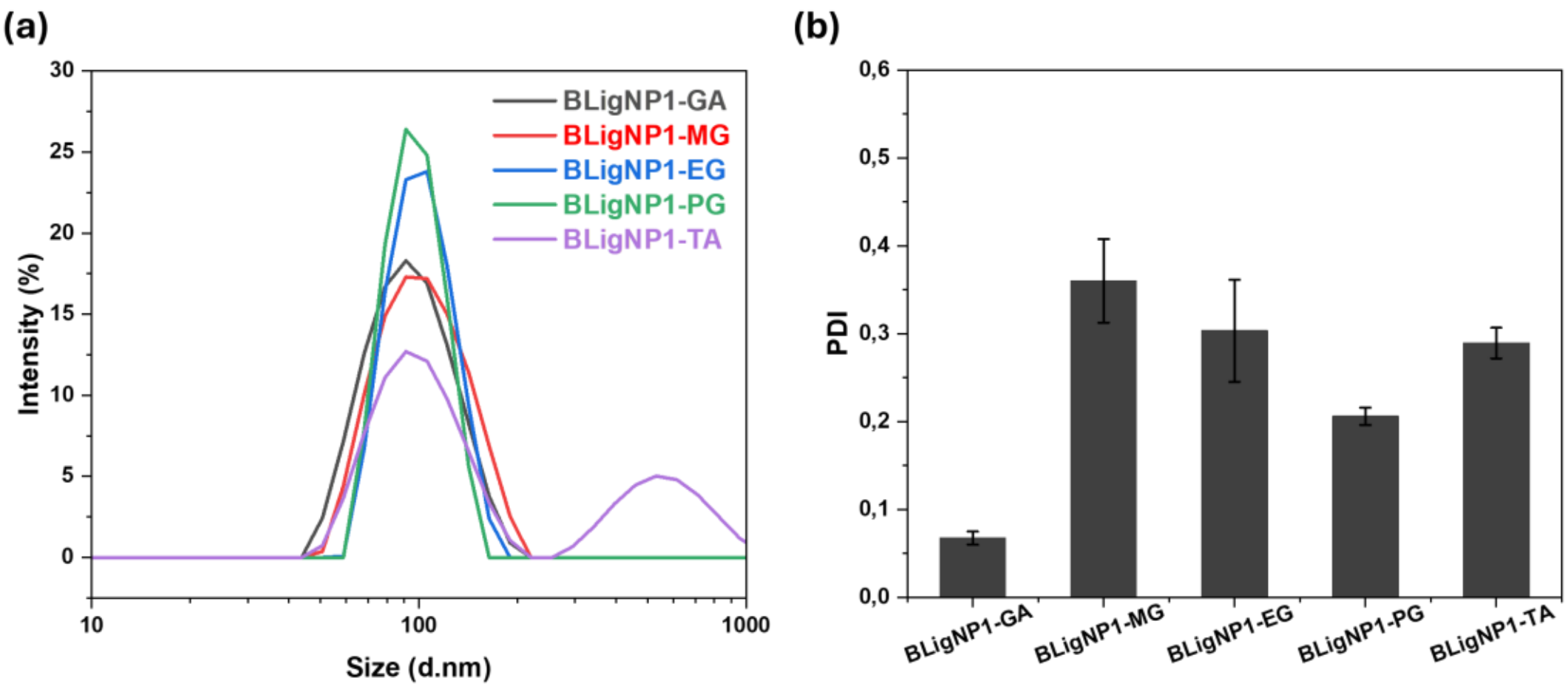

Fig. S8. (a) Hydrodynamic size distribution and (b) PDI of BLigNP1 and BLigNP1-GDs measured by DLS.

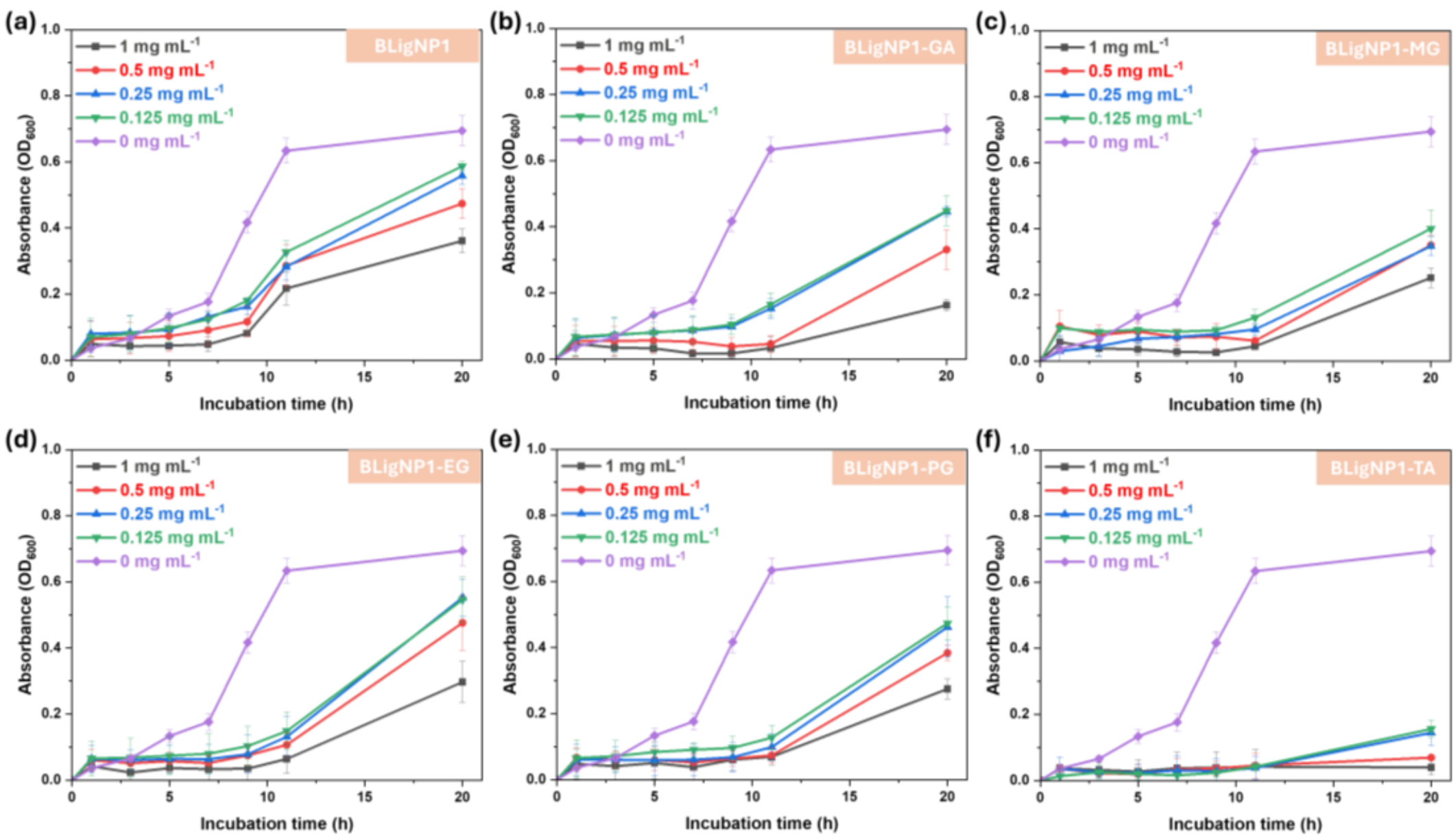

Fig. S9. (a–f) Growth curves of *S. aureus* and upon incubation with different BLigNP-GDs at different concentrations (0–1mg mL^{-1}).

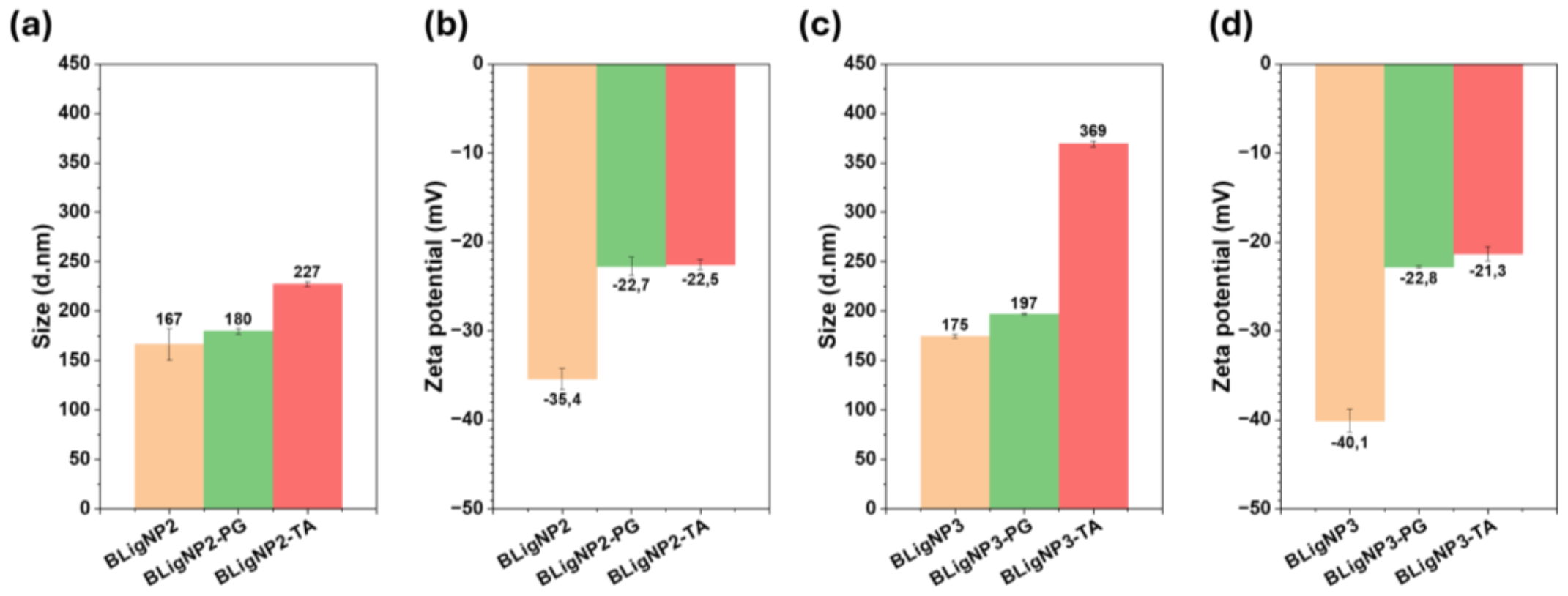

Fig. S10. (a) Hydrodynamic size and (b) zeta potential of BLigNP2, BLigNP2-PG and BLigNP2-TA. (c) Hydrodynamic size and (d) zeta potential of BLigNP3, BLigNP3-PG and BLigNP3-TA.

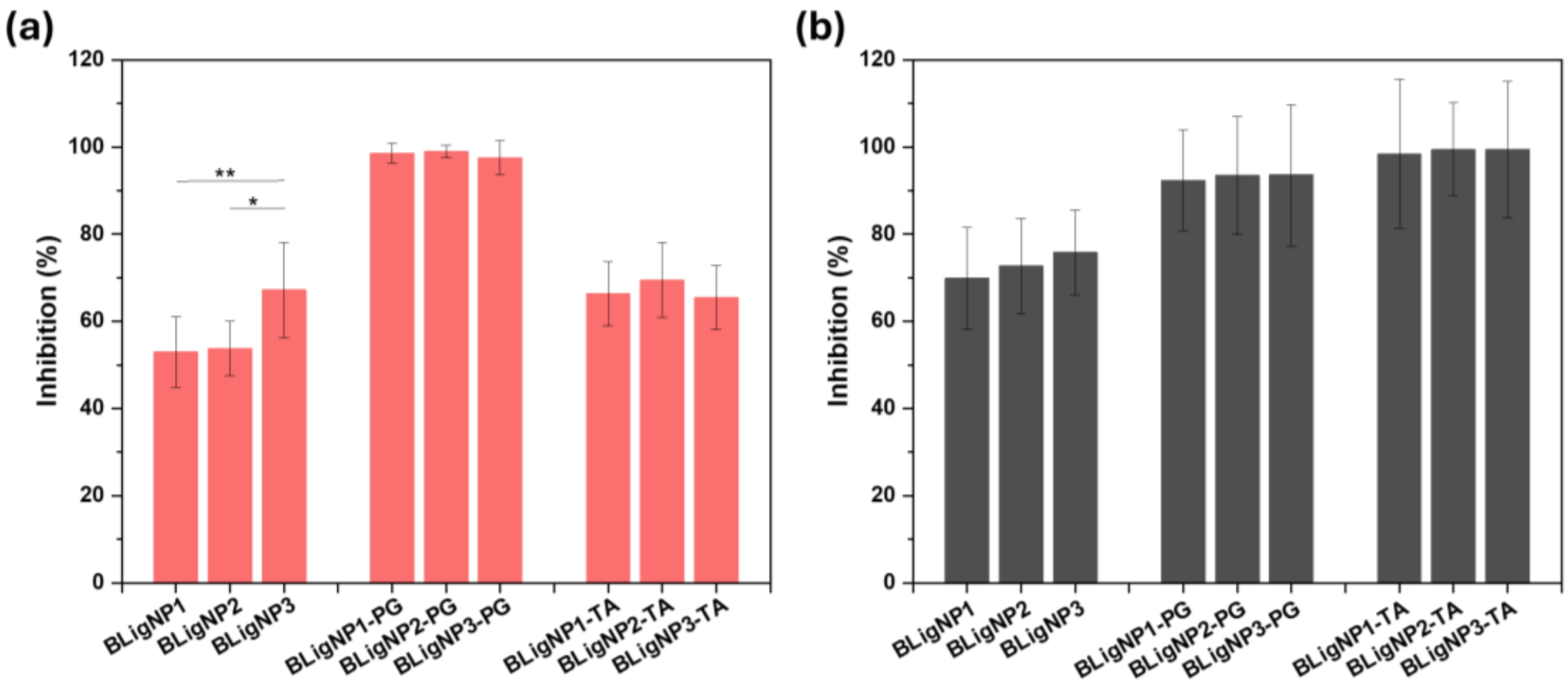

Fig. S11. Growth inhibition of (a) *E. coli* and (b) *S. aureus* after 15h incubation with BLigNPs and BLigNPs-GDs at a concertation of 1 mg mL^{-1}.

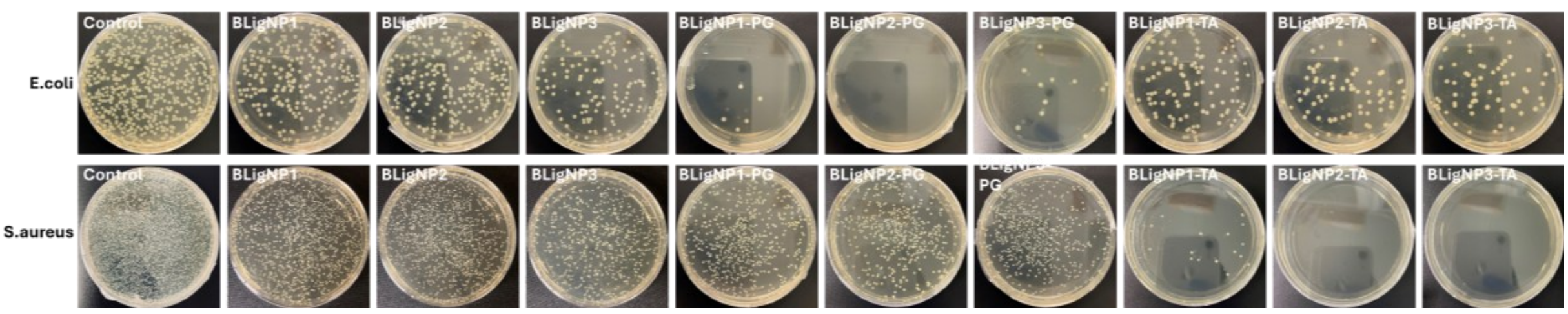

Fig. S12. Digital images of agar-plate showing reduced colony formation after treatment with BLigNPs and BLigNPs-GDs at a concentration of 1 mg mL^{-1} on agar plates.

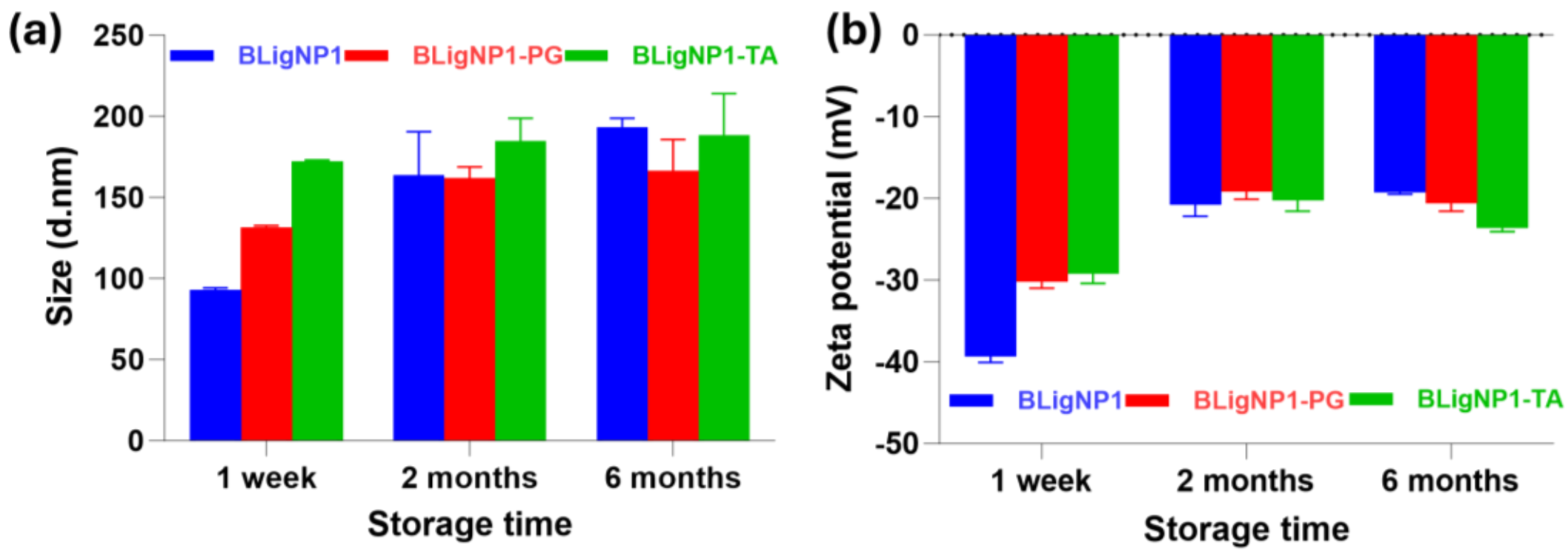

Fig. S13. (a) Hydrodynamic size and (b) zeta potential of BLigNP1, BLigNP1-PG, and BLigNP1-TA as a function of storage time.

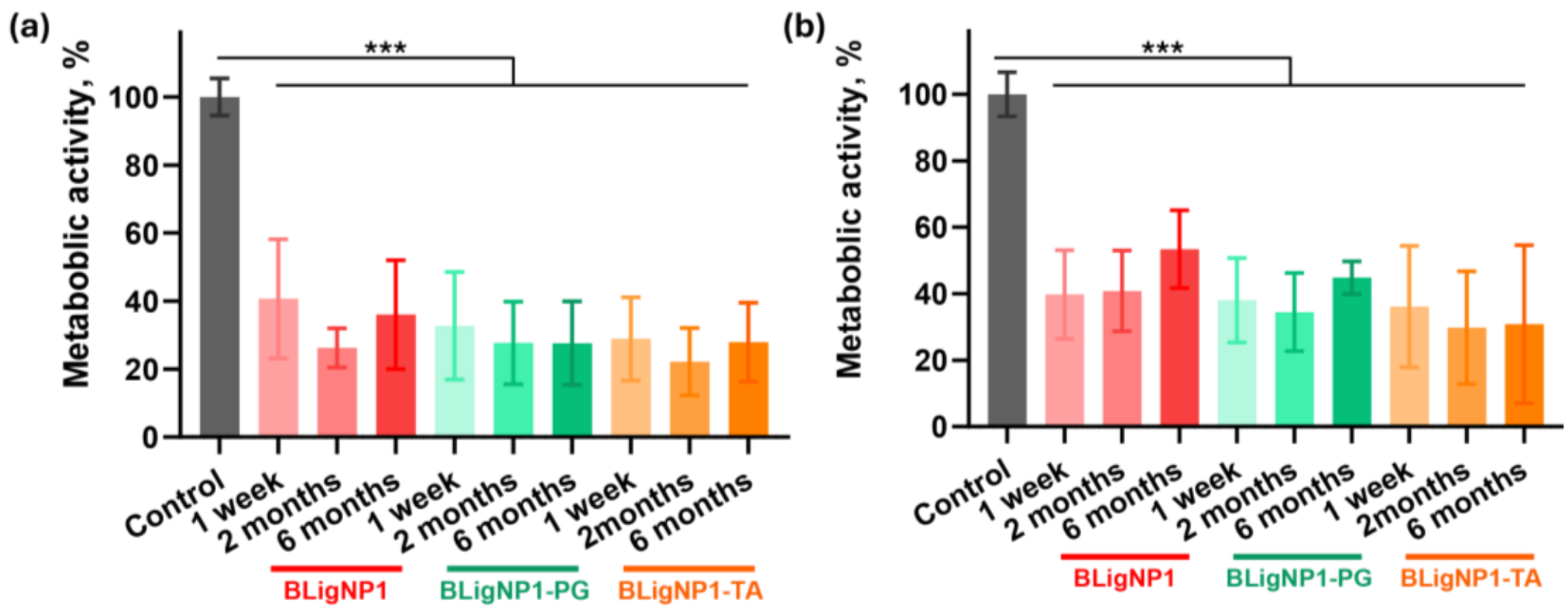

Fig. S14. Antibacterial durability of BLigNP1 and BLigNP1-GDs against (a) *E. coli* and (b) *S. aureus*, reflected by resazurin-based metabolic activity measurements.

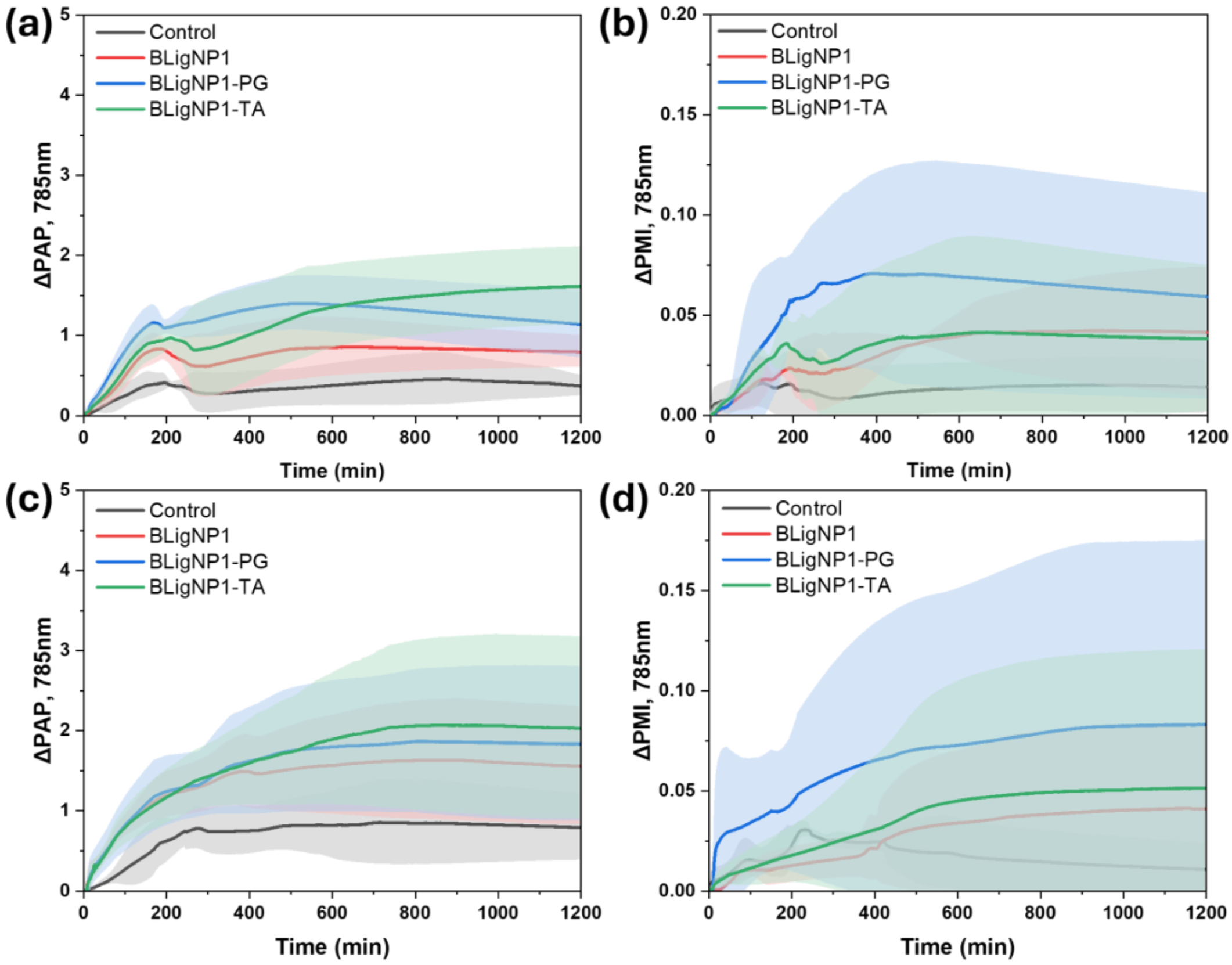

Fig. S15. Average of real-time MP-SPR responses at 785 nm for *E. coli* biofilm under different treatment over 0–1200min: (a) ΔPAP (b) ΔPMI. Average of real-time MP-SPR responses at 785 nm for *S. aureus* biofilm under different treatment over 0–1200min: (c) ΔPAP (d) ΔPMI.

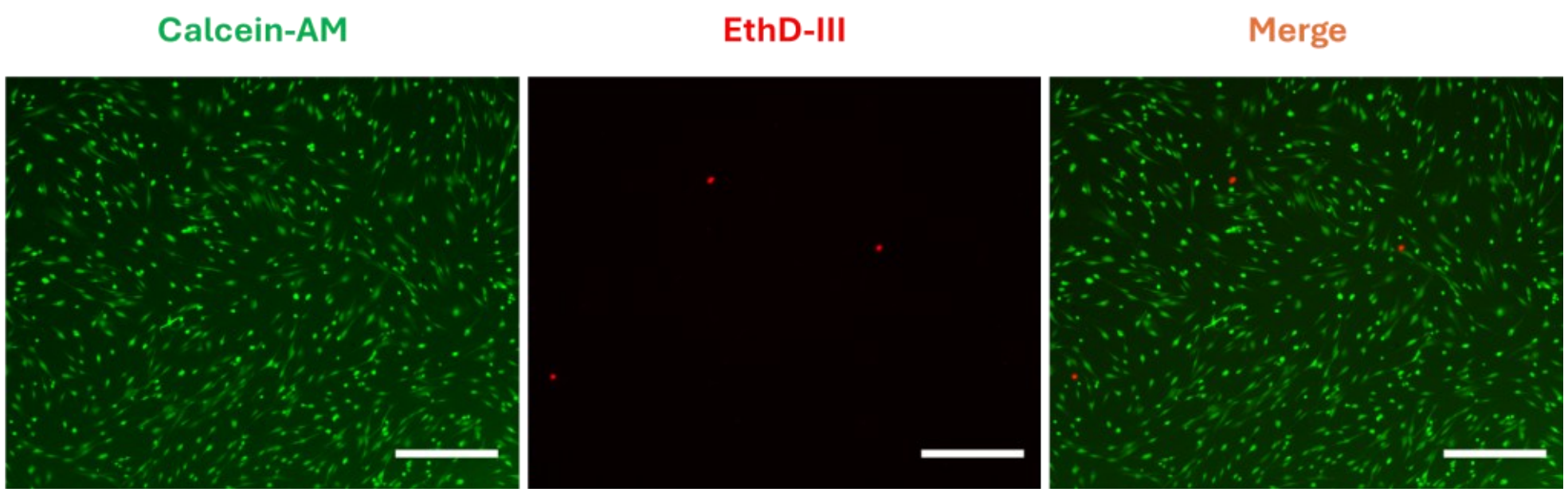

Fig. S16. Live/dead staining (live cells: green; dead cells: red) of untreated HDFs (no polyphenolic NPs exposure). Live cells are shown in green and dead cells in red. Scale bar: 500 µm.

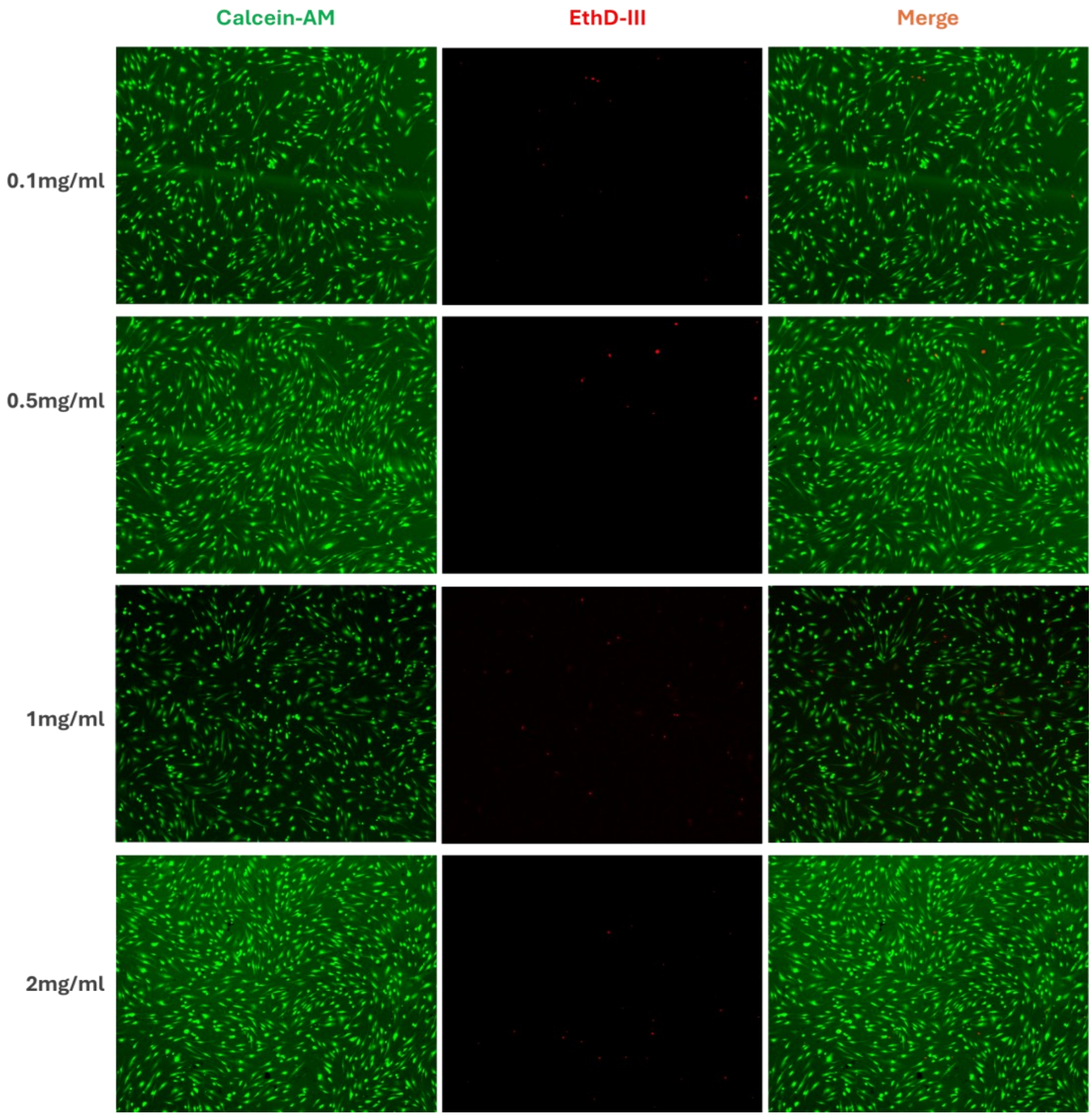

Fig. S17. Live/dead staining (live cells: green; dead cells: red) of HDFs after treatment with BLigNP1 at different concentrations. Live cells are shown in green and dead cells in red. Scale bar: 500 µm.

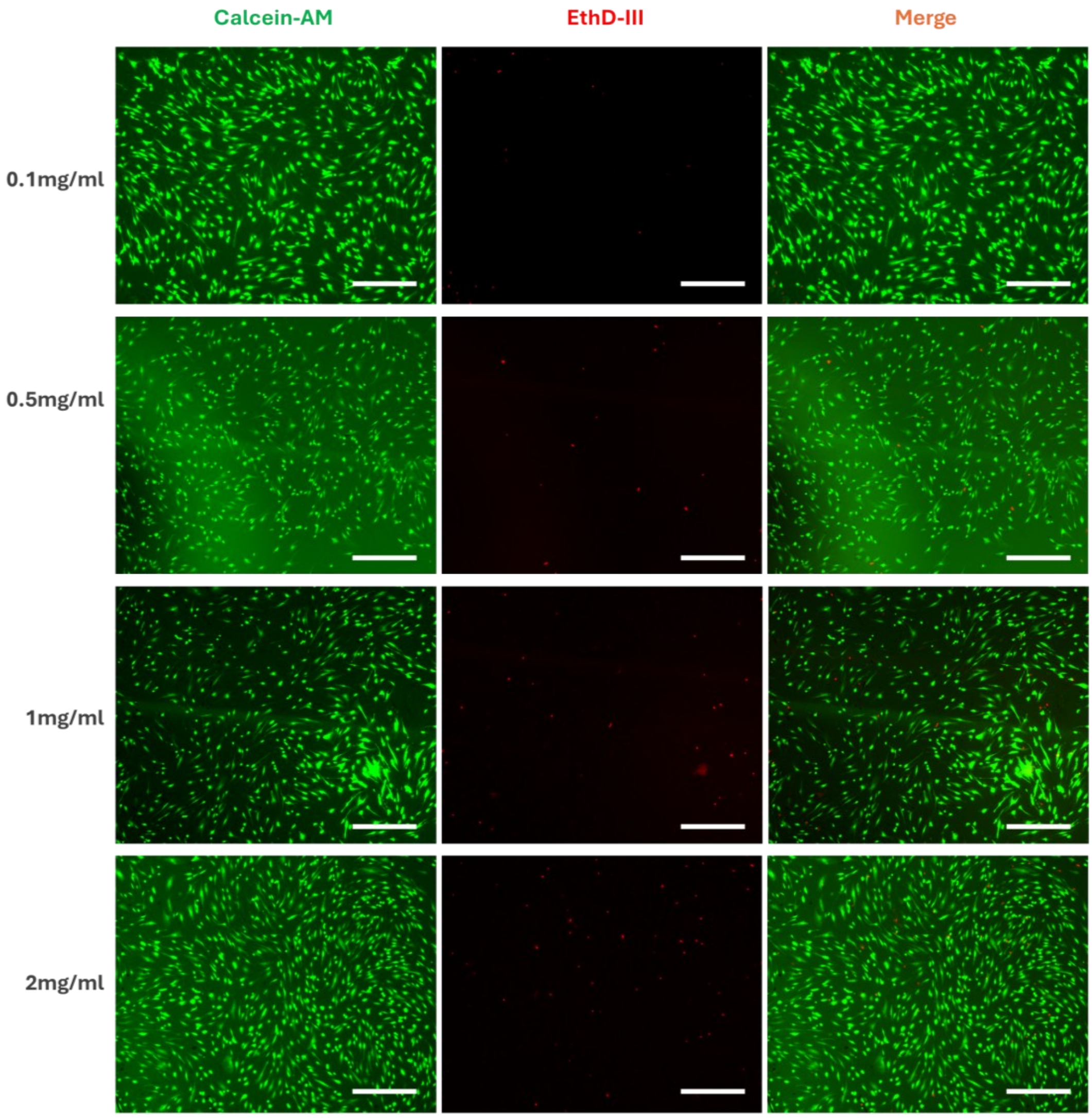

Fig. S18. Live/dead staining (live cells: green; dead cells: red) of HDFs after treatment with BLigNP1-PG at different concentrations. Live cells are shown in green and dead cells in red. Scale bar: 500 μm.

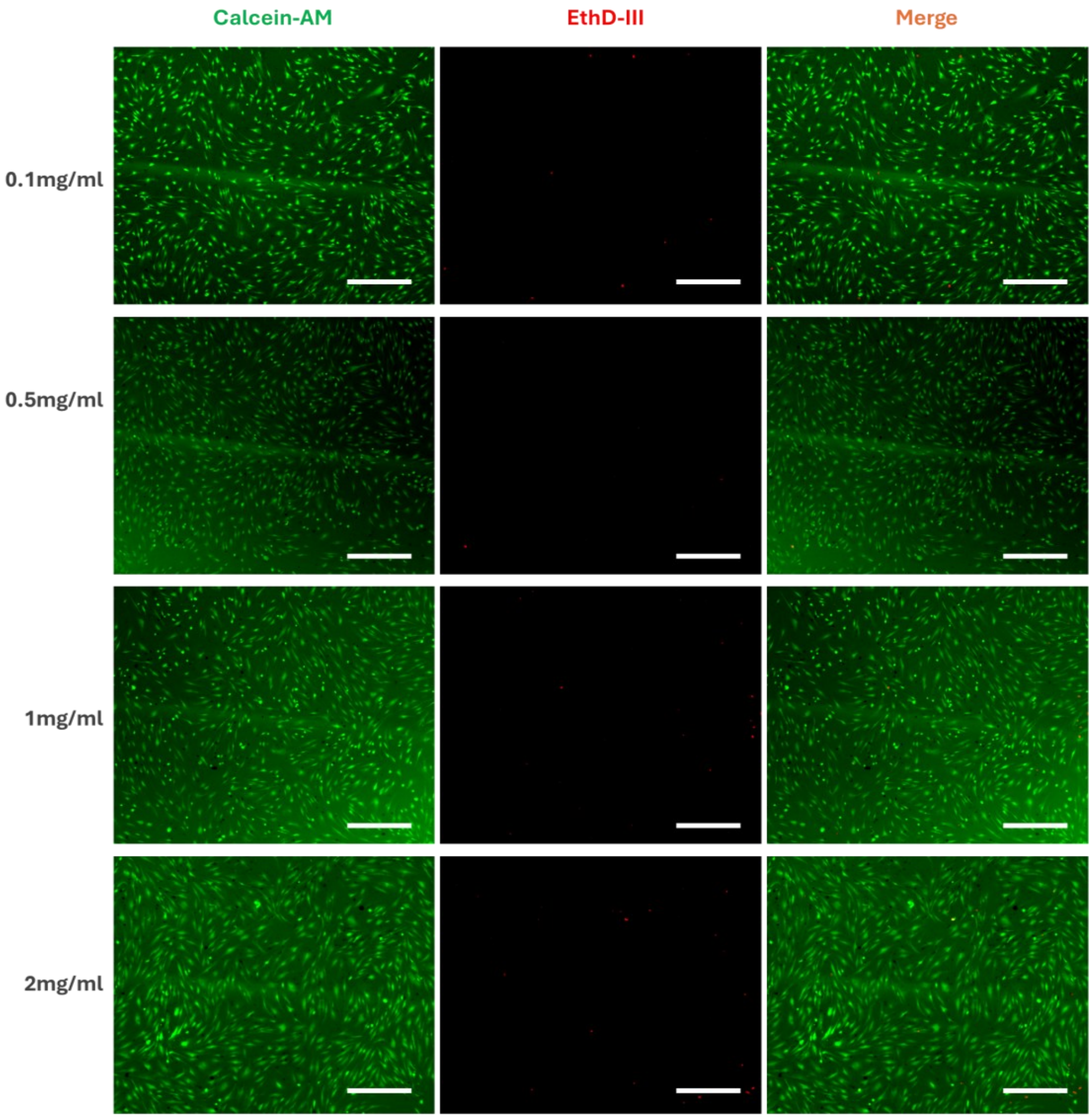

Fig. S19. Live/dead staining (live cells: green; dead cells: red) of HDFs after treatment with BLigNP1-TA at different concentrations. Live cells are shown in green and dead cells in red. Scale bar: 500 μm.

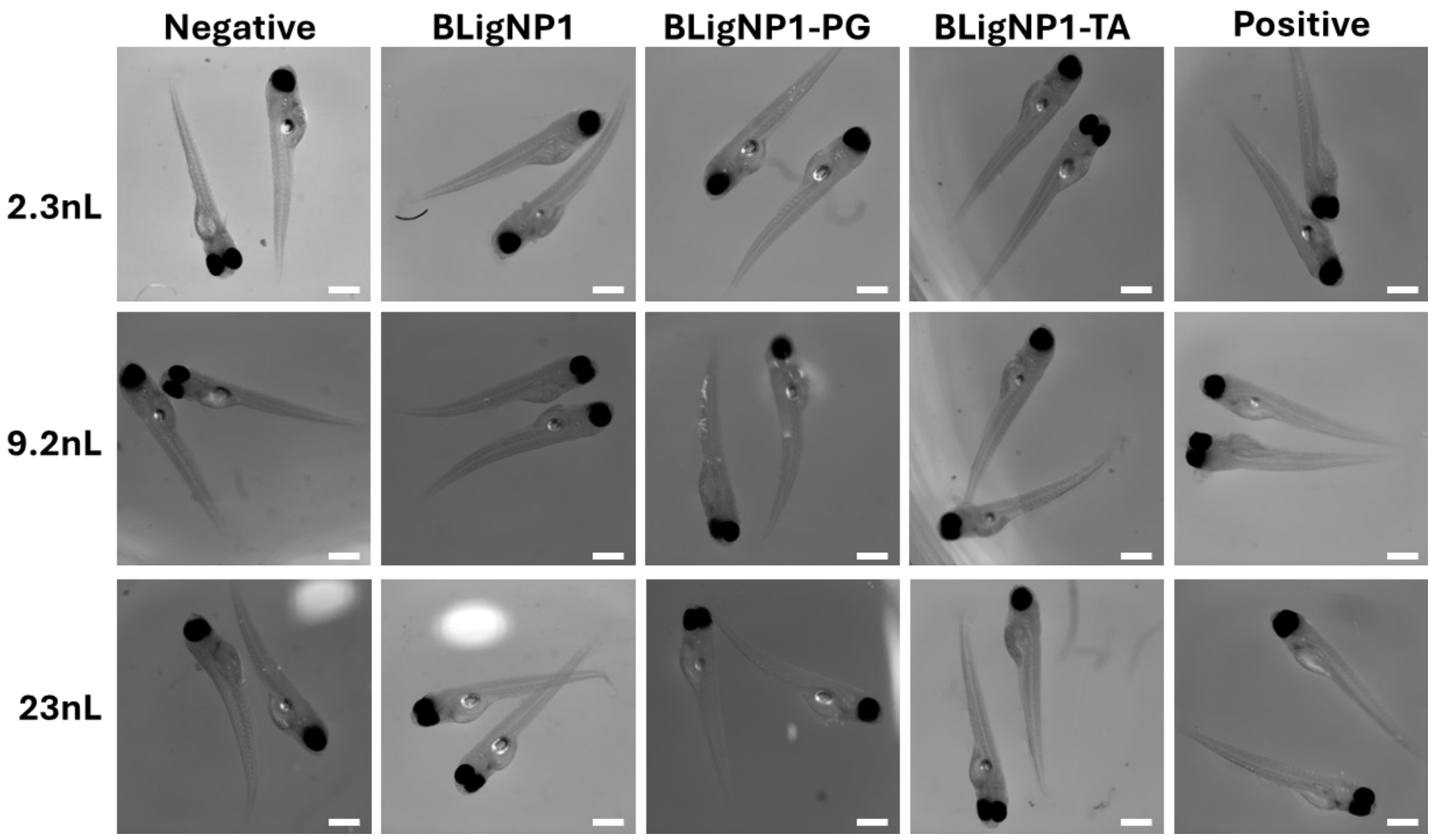

Fig. S20. Representative images of zebrafish embryos injected with BLigNP1 or BLigNP1-GDs (1 mg mL^{-1}) at different injection volumes. Scale bar: 500 µm.

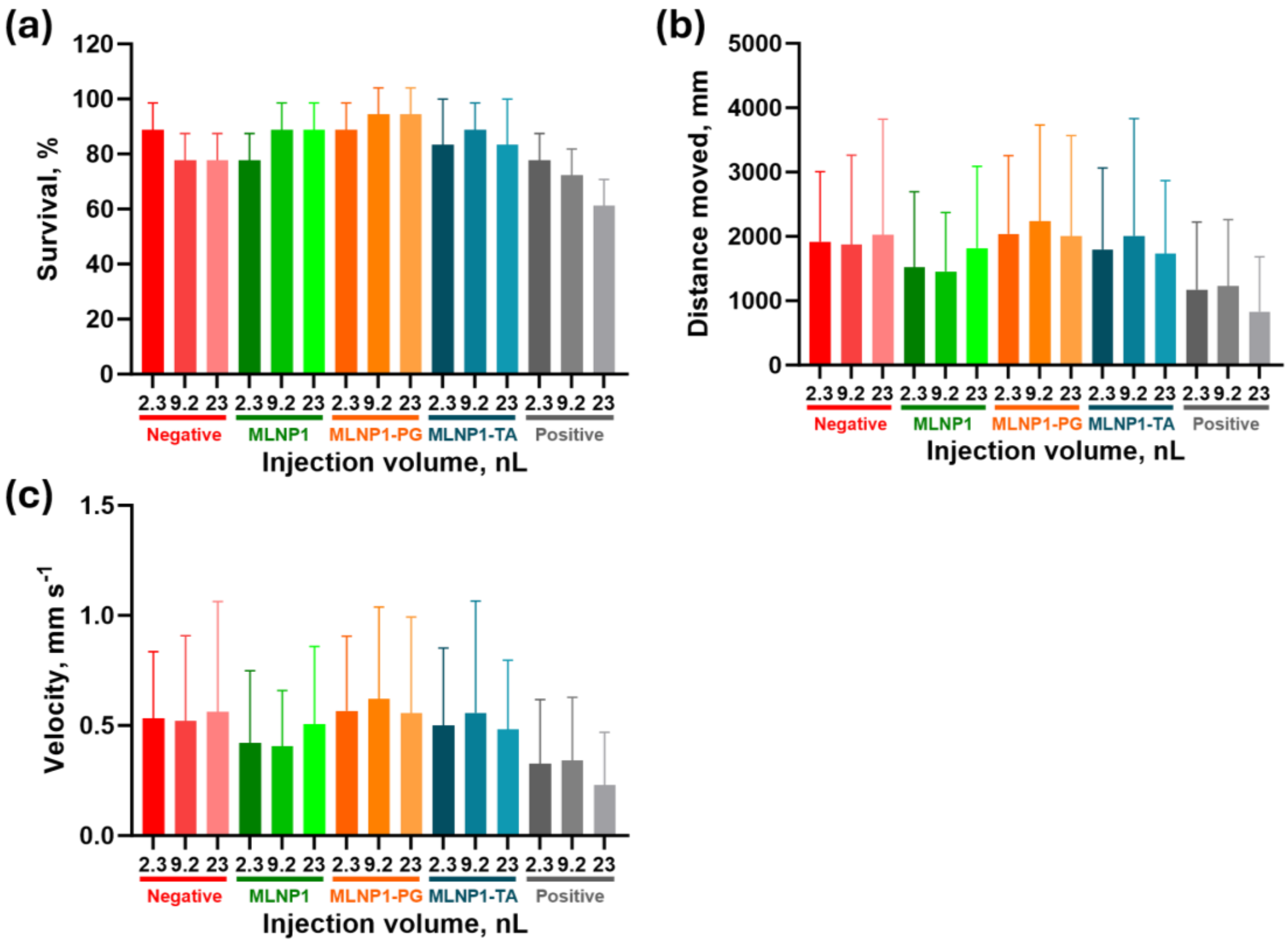

Fig. S21. Locomotor effects of BLigNP1 and BLigNP1-GDs (1 mg mL^{-1}) at different injection volumes, quantified by (a) survival, (b) total distance moved, and (c) swimming velocity.

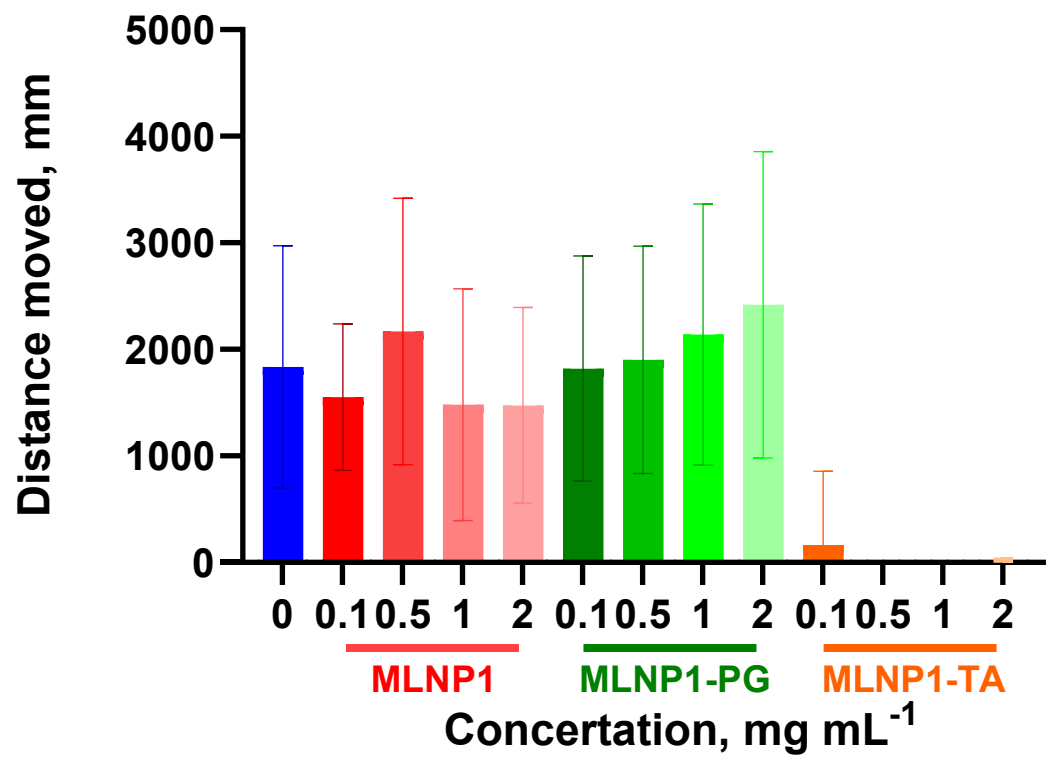

Fig. S22. Total distance moved by zebrafish embryos following exposure to varying concentrations of BLigNP1 and BLigNP1-GDs.

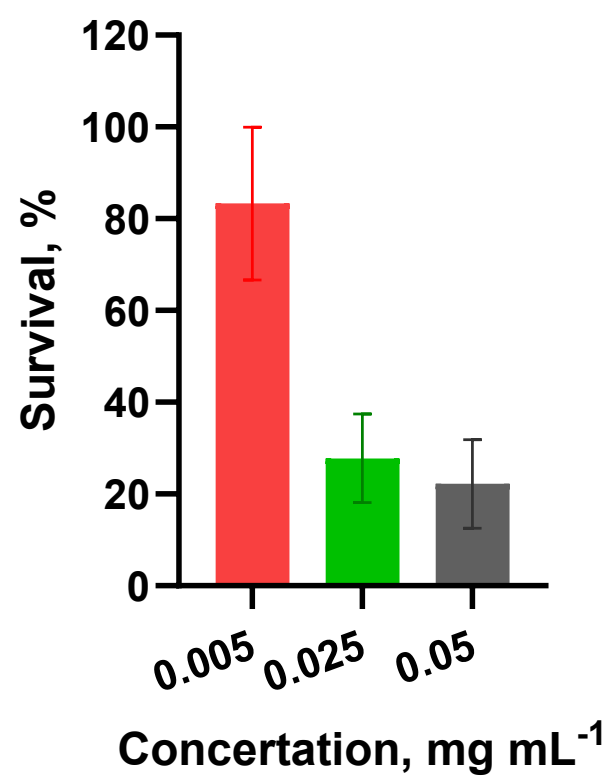

Fig. S23. Survival of zebrafish embryos following exposure to varying concentrations of BLigNP1-TA.

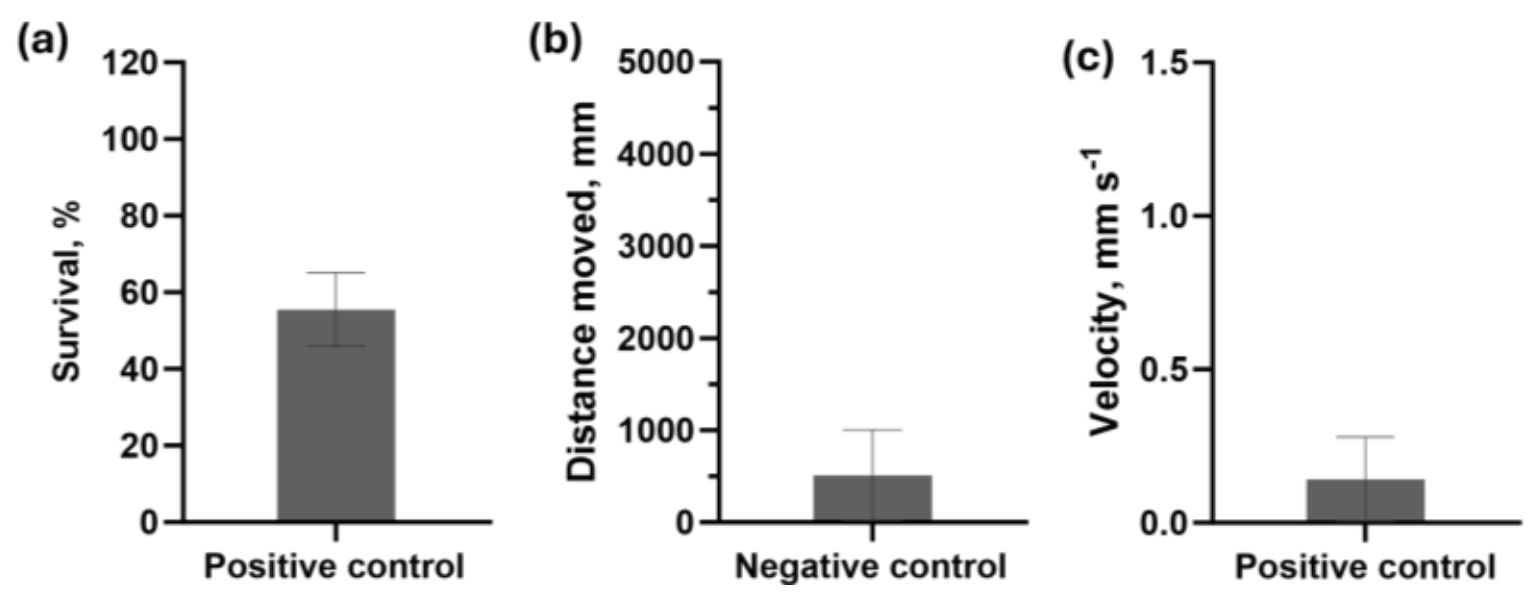

Fig. S24. Effects of the positive control (4 mg L^{-1}, 3,4-dichloroaniline) on zebrafish embryos, quantified by (a) survival, (b) total distance moved, and (c) swimming velocity.

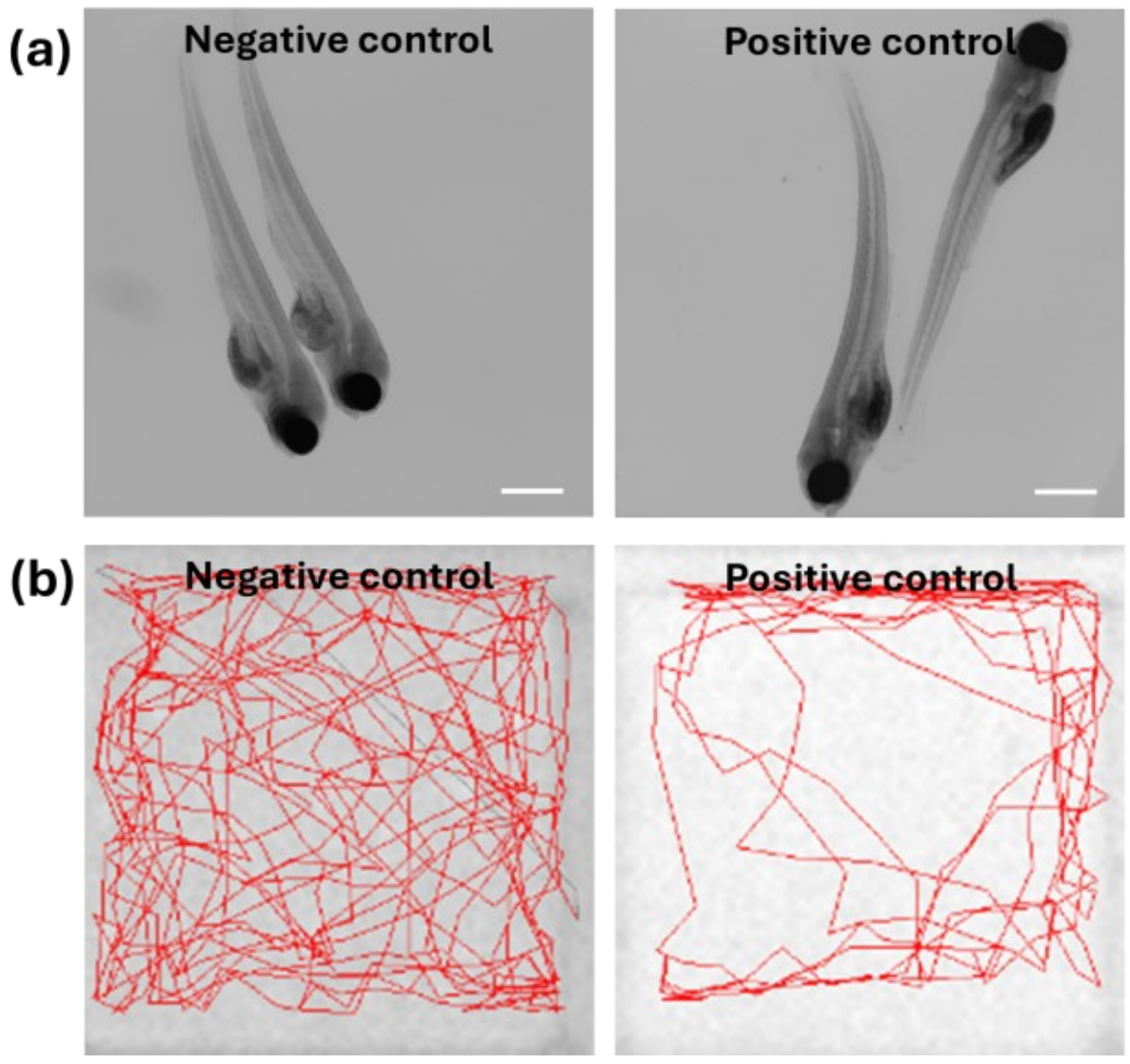

Fig. S25. Representative images showing the effects of the negative control (E3 medium without NPs addition) and the positive control (4 mg L^{-1}, 3,4-dichloroaniline) on zebrafish embryos, assessed by (a) morphology and (b) locomotor trajectories. Scale bar: 500 µm.

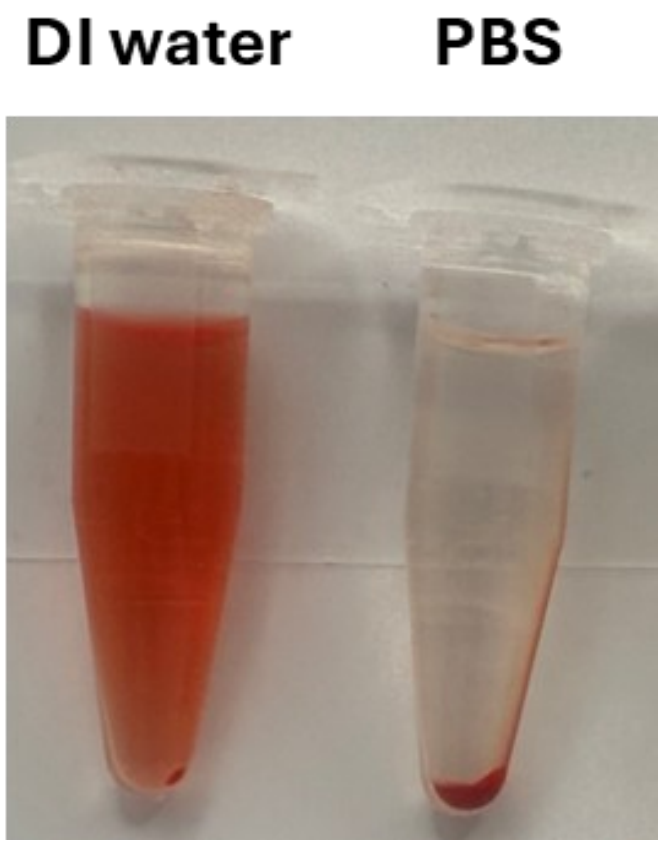

Fig. S26. Digital image of hemolysis controls, with DI water as the positive control and PBS as the negative control.

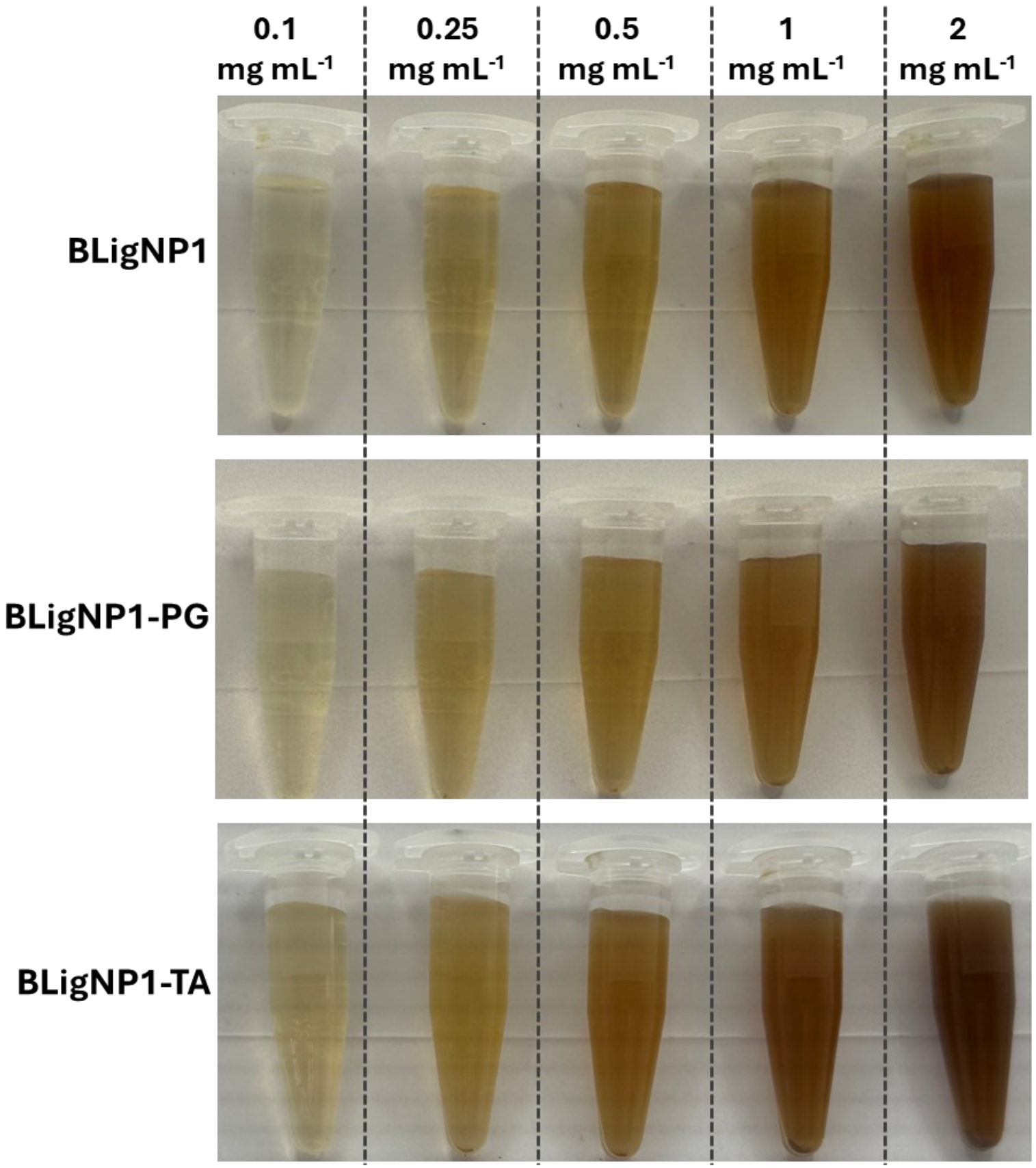

Fig. S27. Digital image of BLigNP1 and BLigNP1-GDs blank control (without RBCs) used in the hemolysis assay.

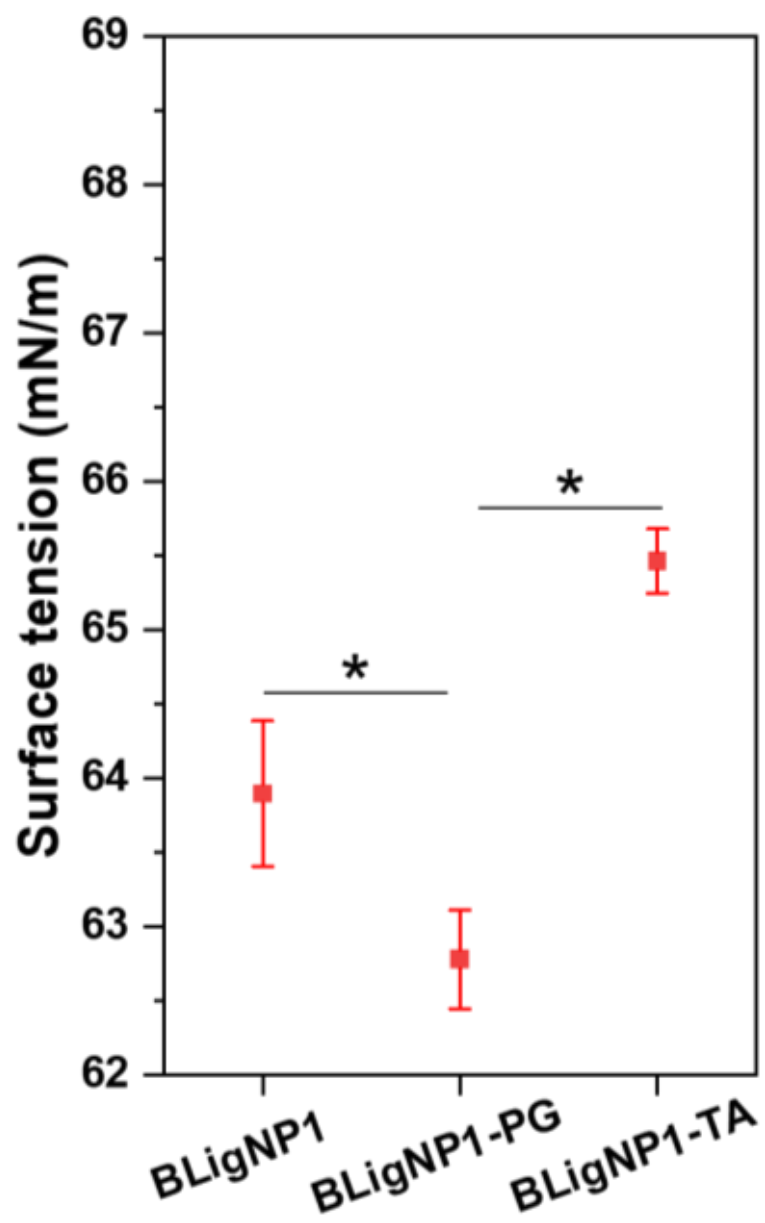

Fig. S28. Air-liquid surface tension of BLigNP1 and BLigNP1-GDs dispersions. For reference, the surface tension of Milli-Q water and PBS was 73.36 ± 0.02 mN m^{-1} and 73.09 ± 0.03 mN m^{-1}, respectively.

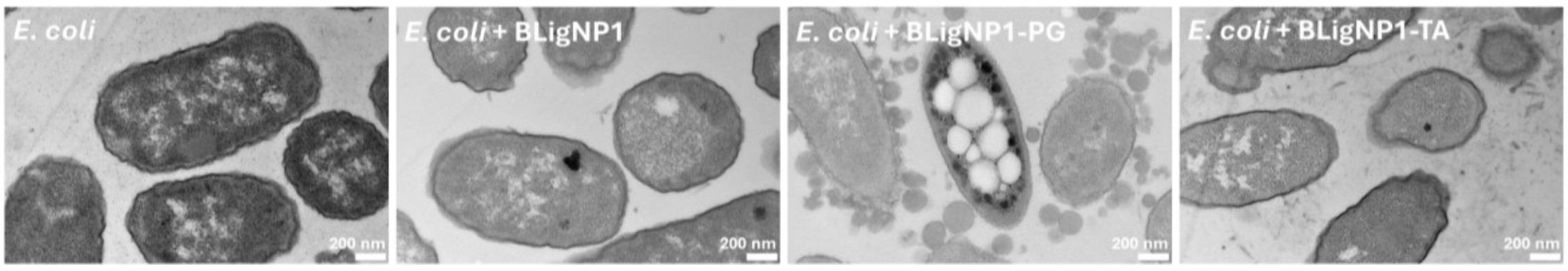

Fig. S29. TEM images of *E. coli* with and without polyphenolic NPs treatment.

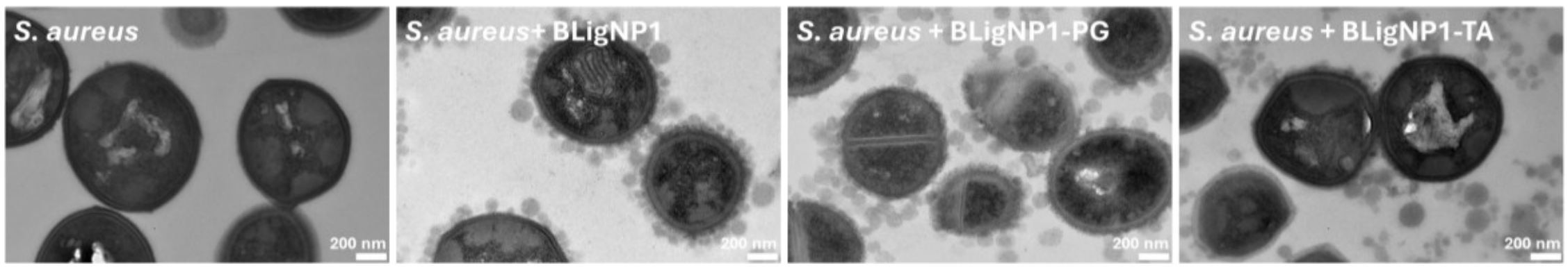

Fig. S30. TEM images of *S. aureus* with and without polyphenolic NPs treatment.

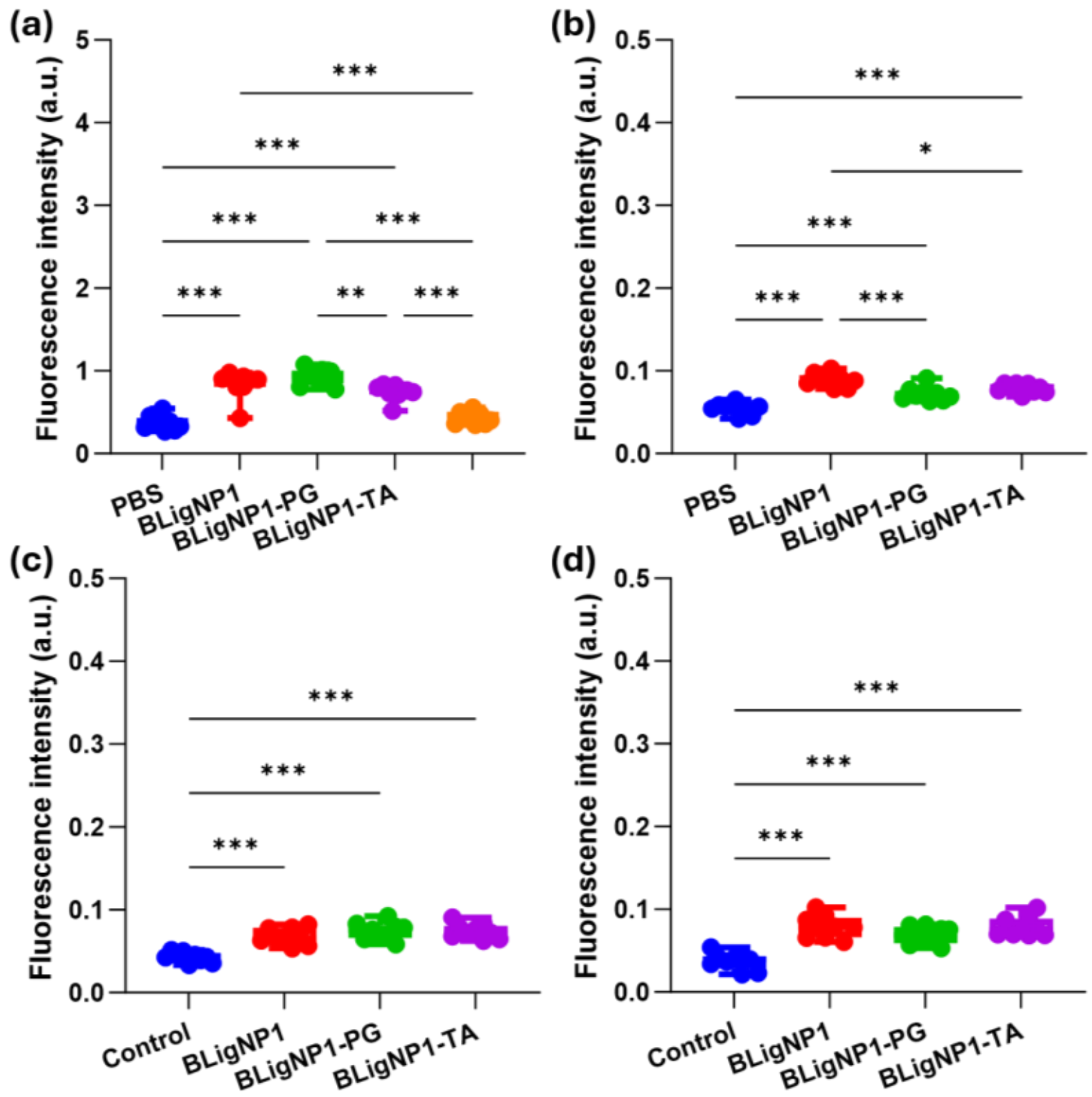

Fig. S31. ROS generation induced by different polyphenolic NPs, quantified by (a) DCFH and (b) DCFH-DA probe in PBS dispersions. Intracellular ROS generation in (c) *E. coli* and (d) *S. aureus* with NPs exposure, measured using the DCFH-DA probe.

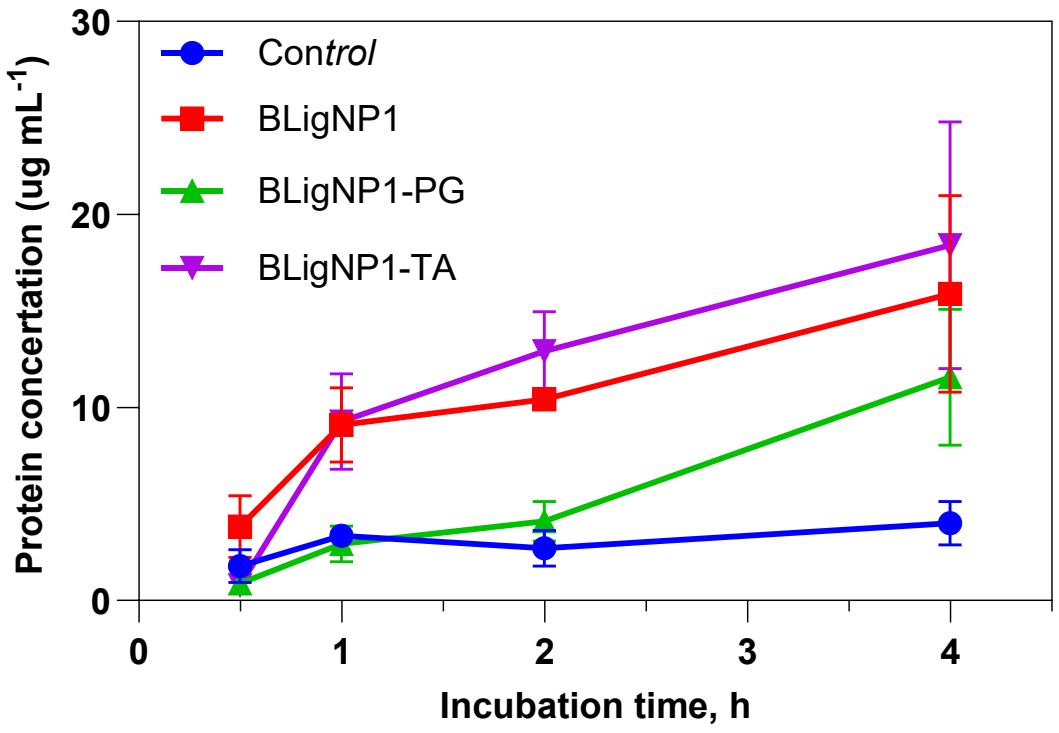

Fig. S32. Protein leakage from *S. aureus* after incubation with BLigNP1, BLigNP1-PG, and BLigNP1-TA.

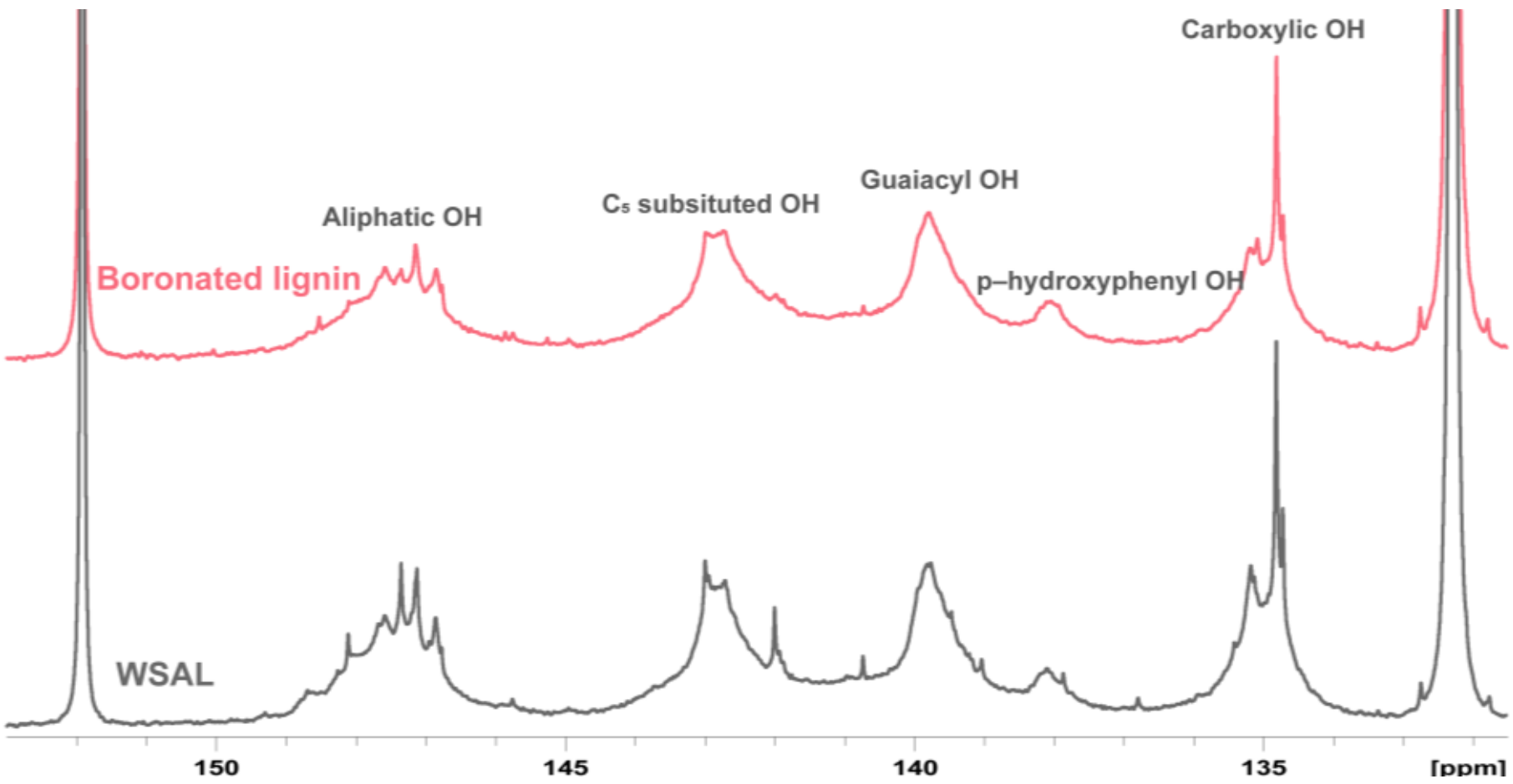

Fig. S33. ^{31}P NMR spectra of WSAL and boronated lignin showing the distribution of hydroxyl group.

Table S1. Molar mass and dispersity of lignin powder (WSAL) and BLigNP with different APBA grafting density.

	Lignin powder	BLigNP1	BLigNP2	BLigNP3
M_n (kDa)	3.05	4.30	4.90	7.50
M_w (kDa)	18.03	32.82	38.71	38.92
$Đ_M$	5.16	7.59	7.94	5.22

$Đ_M$: molar mass dispersity (M_w/M_n)

Table S2. The amount of OH groups of lignin powder and BLigNP with different APBA grafting density.

	Lignin powder	BLigNP1	BLigNP2	BLigNP3
Aliphatic OH, mmol/g	1.28	1.13	1.06	1.02
C_5 substituted OH, mmol/g	1.74	1.51	1.49	1.44
Guaiacyl OH, mmol/g	0.91	0.78	0.78	0.75
p-hydroxyphenyl OH, mmol/g	0.55	0.48	0.49	0.47
Total phenolic OH, mmol/g	3.20	2.77	2.76	2.66
Carboxylic acid OH, mmol/g	1.02	0.83	0.81	0.78

REFERENCES

[1] Z. Lu, J. Tienaho, P. Kilpeläinen, M. Hummel, H. Sixta, C. Xu, Laccase-catalyzed copolymerization of tannin and technical lignin: polymerization kinetics and enhanced properties of polyphenols, ACS Sustain. Chem. Eng. 12 (2024) 8765–8779.

[2] L. Wang, L. Tan, L. Hu, X. Wang, R. Koppolu, T. Tirri, B. van Bochove, P. Ihalainen, L.S. Seleenmary Sobhanadhas, J.V. Seppälä, S. Willför, M. Toivakka, C. Xu, On laccase-catalyzed polymerization of biorefinery lignin fractions and alignment of lignin nanoparticles on the nanocellulose surface via one-pot water-phase synthesis, ACS Sustain. Chem. Eng. 9 (2021) 8770–8782.

[3] R.A. Mustafa, P. Parkkila, J.M. Rosenholm, H. Zhang, T. Viitala, Monitoring silica core@shell nanoparticle-bacterial film interactions using the multi-parametric surface plasmon resonance technique, Smart Med. 2 (2023) e20230012.
